# Preservation and Reuse of KLEx Lenticules: From Biobanking to Therapeutic and Refractive Innovations

**DOI:** 10.3390/bioengineering13091075

**Published:** 2026-09-16

**Authors:** Raimy Christodoulou, Stefano Ferrari, Diego Ponzin, Mario Nubile, Andri K. Riau, Jodhbir S. Mehta

**Affiliations:** 1 The Veneto Eye Bank Foundation, 30174 Venice, Italystefano.ferrari@fbov.it (S.F.); diego.ponzin@fbov.it (D.P.); 2 Regenerative Therapy Research Group, Singapore Eye Research Institute, Singapore 169856, Singapore; 3 University of Ferrara, 44121 Ferrara, Italy; 4 Department of Translational Medicine, University of Ferrara, 44121 Ferrara, Italy; 5 Ophthalmology Clinic, Department of Medicine and Aging Science, “G. d’Annunzio” University of Chieti-Pescara, 66100 Chieti, Italy; 6 Singapore National Eye Centre, Singapore 168751, Singapore

**Keywords:** keratorefractive lenticule extraction, lenticule biobanking, stromal keratophakia, ocular drug delivery, tissue engineering, additive keratoplasty

## Abstract

Stromal keratophakia was first introduced by Jose Ignacio Barraquer in the 1960s as a refractive procedure involving the implantation of a stromal lenticule into a lamellar keratectomy bed of a recipient cornea. By increasing central corneal thickness, the added lenticule induced steepening of the anterior corneal curvature, thereby increasing the refractive power. However, due to the technical limitations of the mechanical microkeratome and the use of cryolathed tissue, early clinical outcomes were often inconsistent and included risks of stromal necrosis, epithelial ingrowth, induced astigmatism, slow visual recovery, and corneal haze. Despite these limitations, Barraquer’s law of thickness laid the groundwork for modern refractive surgery, including Laser-In Situ Keratomileusis (LASIK). The advancement of femtosecond laser technology has enabled the creation of precise and accurate refractive lenticules, stromal flaps, and pockets. This, in turn, has renewed interest in stromal keratophakia and facilitated the emergence of keratorefractive lenticule extraction (KLEx) procedures, through which stromal lenticules are obtained. These lenticules can be preserved and repurposed for various therapeutic and refractive applications. In this review, we provide a comprehensive overview of KLEx-derived lenticule processing and biobanking, their reuse for hyperopia and presbyopia correction, and their therapeutic potential in stromal thickness restoration, cell-based tissue engineering, drug delivery, corneal structural and macular hole repair, and glaucoma drainage tube coverage.

## 1. Introduction

The total refractive power of the human cornea is approximately 43 diopters, accounting for two-thirds of the total optical eye power, with the remaining third provided by the lens [1]. As corneal refractive power is dependent on the curvature [2], the early surgical approach focused on altering the corneal shape with the aim of correcting refractive errors, such as myopia, hyperopia, and astigmatism [3,4,5].

**Figure 1:**
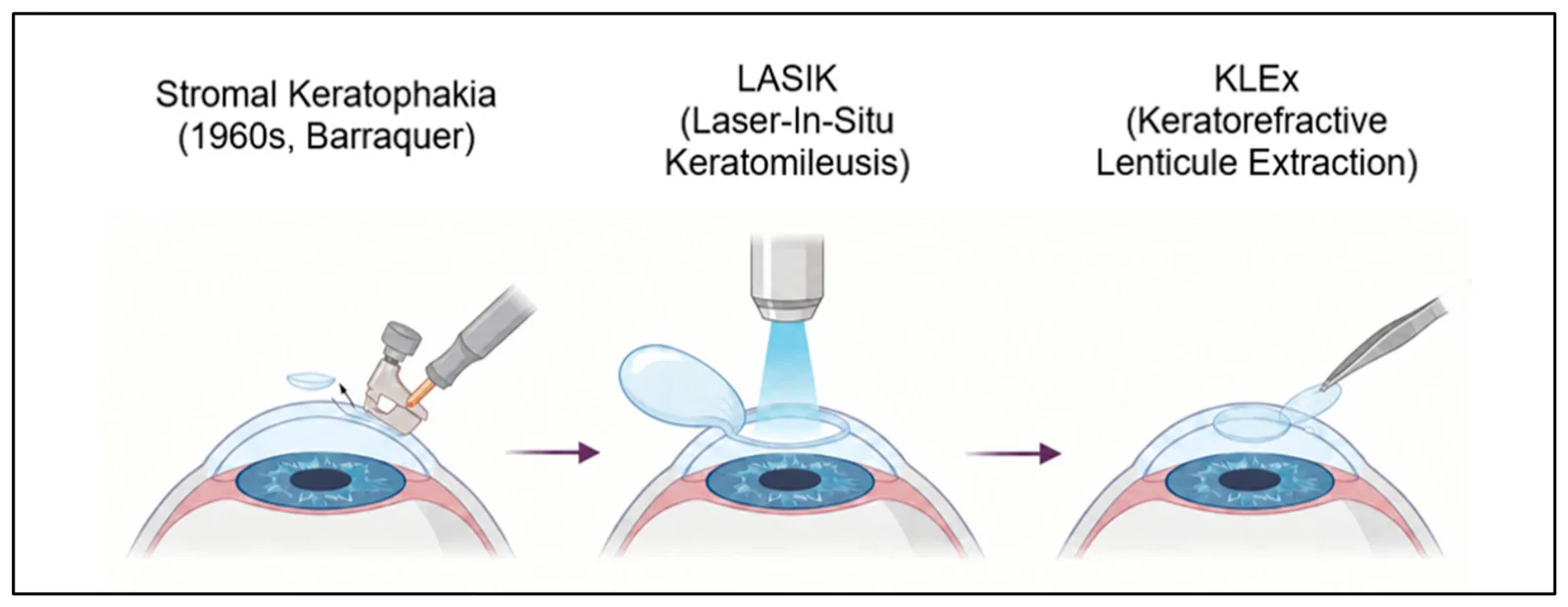
Illustration of keratorefractive procedures. Stromal keratophakia, first introduced by Jose Ignacio Barraquer in the 1960s, is an additive corneal procedure involving the implantation of a stromal lenticule into a lamellar keratectomy of a recipient cornea. Technical limitations of the mechanical microkeratome and the use of cryolathed tissue resulted in inconsistent early clinical outcomes [6,8,9,10,11,12]. Barraquer’s Law of Thickness laid the groundwork for modern refractive surgery, including Laser-In Situ Keratomileusis (LASIK), which significantly improved the safety, predictability and recovery compared to earlier techniques [13]. Subsequent advances in femtosecond laser technology enabled the creation of precise refractive lenticules, ultimately facilitating the rise in keratorefractive lenticule extraction (KLEx) procedures [17]. Created in BioRender. Barbaro, V. (2026) https://BioRender.com/j1nzj6t. (accessed on 1 September 2026).

Stromal keratophakia, first introduced by Jose Ignacio Barraquer in the 1960s, is a corneal additive procedure involving the implantation of a fresh or preserved stromal lenticule into a lamellar keratectomy of a recipient cornea. By increasing central corneal thickness, the added lenticule induced steepening of the anterior corneal curvature, thereby increasing the refractive power of the cornea [6,7,8]. However, due to the technical limitations of the mechanical microkeratome and the use of cryolathed tissue, early clinical outcomes were often inconsistent and included risks of stromal perforation, necrosis, epithelial ingrowth, induced astigmatism, slow visual recovery, and corneal haze [6,8,9,10,11,12]. Despite these limitations, Barraquer’s Law of Thickness laid the groundwork for modern refractive surgery, including Laser-In Situ Keratomileusis (LASIK) [13] (Figure 1).

In an attempt to overcome the risk of corneal perforation induced by the mechanical microkeratome during stromal keratophakia, Herbert Kaufman and Theodore Werblin introduced epikeratophakia [14]. During this procedure, the lenticule is placed and sutured directly on the anterior surface of the recipient cornea, after the removal of the epithelial layer [11]. In this technique, a peripheral annular keratectomy/groove was performed to allow the lenticule to be secured to the recipient cornea with sutures. Nevertheless, clinical outcomes were unpredictable and included interface irregularities, epithelial ingrowth, and prolonged visual recovery [15,16]. As a result, both stromal keratophakia and epikeratophakia were abandoned.

The advancement in femtosecond laser technology has enabled the creation of precise refractive lenticules, stromal flaps, and pockets [17]. This, in turn, has renewed interest in stromal keratophakia and facilitated the rise in keratorefractive lenticule extraction (KLEx) procedures [18]. During KLEx surgery, the extracted lenticule is generally discarded as medical waste [17]. However, with the increasing number of these procedures [19] and the shortage of donor corneal tissue, especially in low-resource regions [20], these lenticules represent a valuable source of viable stromal tissue with potential for reuse in a range of corneal reconstructive procedures [21]. Indeed, the native collagen architecture, optical transparency, mechanical strength, and biocompatibility make KLEx-derived lenticules particularly suitable for tissue engineering and transplantation. Furthermore, KLEx-derived lenticules are often derived from young, healthy donors, making them suitable for various therapeutic applications [22].

The concept of reusing KLEx-derived lenticules was first validated in animal models, initially in rabbits and subsequently in non-human primates, by Angunawela et al. and Riau et al., who demonstrated that KLEx-derived lenticule implantation can effectively restore stromal volume [23,24]. Soon after, the concept was translated into clinical practice in multiple studies that reported repurposing of KLEx-derived lenticules, particularly for hyperopia and presbyopia correction, corneal thickness restoration in keratoconus, creation of scleral patches, and even as scaffolds for ocular drug release and cell-based regenerative therapies [25,26,27,28,29].

Three previous reviews provide the immediate context for the present update [11,21,30]. The present review is intended to complement rather than duplicate these works. Relative to Riau et al. and Bievel-Radulescu et al., we additionally incorporate a body of work that has matured only in the past five years: the establishment of the first regulator-licenced corneal lenticule biobank, the progression of stromal lenticule addition keratoplasty for keratoconus toward biomechanical and individualized cone-geometry-matched approaches, the emergence of decellularized lenticules as a sustained-release drug delivery platform, the first posterior-segment applications in refractory macular hole and macular hole retinal detachment, and the regulatory advance of acellular lenticule-derived corneal allografts toward FDA Investigational Device Exemption. Whereas Zhang et al. focused primarily on preservation methodologies, we frame the lenticule as the central biological asset of an end-to-end biobanking workflow, encompassing donor screening, quality assurance, regulatory classification, and distribution alongside preservation. Together, these developments mark the transition of KLEx lenticule reuse from a collection of innovative case reports into a structured therapeutic platform.

Similar to corneal transplantation, the widespread use of KLEx-derived lenticules depends largely on effective tissue processing and long-term storage [31]. Therefore, a standardized preservation technique is essential for ensuring optimal surgical outcomes and minimizing the risk of complications. In this context, this review aims to provide a comprehensive overview of KLEx-derived lenticules, addressing current processing and preservation strategies, their refractive application in hyperopia and presbyopia correction, and their innovative therapeutic applications in corneal structural disorder and ocular repair.

## 2. Lenticule Banking

The immediate use of fresh KLEx lenticules is logistically challenging when lenticules are obtained from refractive surgery patients, as it requires coordination of two surgical procedures (refractive lenticule extraction and intrastromal keratoplasty), which may not always be feasible. Therefore, establishing a lenticule bank for collection, processing, preservation, and distribution is essential to enhance the clinical accessibility of lenticules.

### 2.1. Short-Term Storage and Transportation

**Figure 2:**
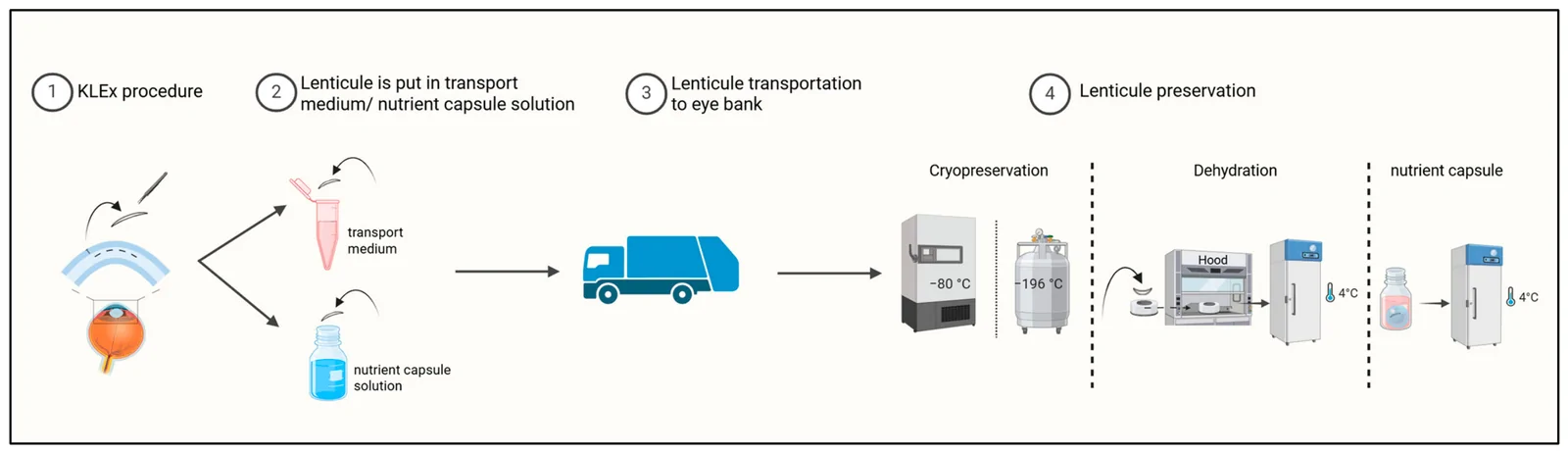
Graphical overview of KLEx lenticule banking. Directly after extraction, lenticules are put either in a transport medium or a nutrient capsule solution and transported to the eye bank. Potential preservation techniques include cryopreservation, dehydration, or storage in a nutrient capsule. Figure created in BioRender. Barbaro, V. (2026) https://BioRender.com/s3fvzuq. (accessed on 1 September 2026).

Most refractive centres lack direct access to laboratory facilities required for long-term preservation. Therefore, there is a need for an intermediate, inexpensive transport medium that is easily accessible as a transient carrier before longer storage (Figure 2). Liu et al. evaluated various solutions, including phosphate-buffered saline (PBS), Optisol-GS (Bausch & Lomb Inc., Rochester, NY, USA) (a standard corneal preservation medium), Dulbecco’s Modified Eagle’s Medium (DMEM) with fetal bovine serum (FBS), and anhydrous glycerol at either 4 °C or room temperature. Their findings indicated that all four media effectively maintained lenticule clarity for up to 48 h, with only minor, non-significant changes in spectral transmittance [32]. Recently, our group has further demonstrated that hyaluronic acid (HA) is a promising transport medium, as it preserves the physiological stromal architecture of KLEx lenticules more effectively compared to Coldix, a dextran-based MEM medium commonly used in the eye bank [33]. Beyond the choice of medium, the duration of transportation is a key factor and should also be taken into consideration. A study by Soo et al. indicated that lenticules stored in PBS can maintain transparency for up to 5 days at 4 °C. Further delays compromised ultrastructural integrity and postoperative clarity, emphasizing the importance of integrating transportation logistics into banking protocols [34].

### 2.2. Long-Term Preservation Techniques

Cryopreservation is the most common technique used for long-term storage, allowing tissue to be stored at temperatures between −80 °C and −196 °C for an extended period of time without significant structural alteration [24,35,36]. Cellular preservation is typically achieved using cryoprotectant agents like dimethyl sulfoxide (DMSO). However, it negatively affects the stromal extracellular matrix by disrupting native collagen fibril organization and compromising biomechanical integrity [33]. An alternative to this is preservation in anhydrous glycerol, which offers excellent dehydrating and antimicrobial properties and is well-established for lamellar corneal tissues (Table 1) [37,38]. While glycerol effectively maintains collagen integrity, it does not preserve stromal keratocyte viability. This, however, is not an overriding concern, as migration of recipient keratocytes would be expected in the longer term [38,39]. Nonetheless, the biological relevance of preserving keratocyte viability depends on the clinical application. For refractive implantation, including hyperopic and presbyopic correction, viable keratocytes within the lenticule are not essential, as the primary objective is to modify the corneal stromal volume and curvature. Similarly, when KLEx lenticules are used for tectonic keratoplasty, their main function is to provide tissue coverage and structural support. Although viable cells may contribute to tissue remodelling, regenerative response and repopulation, preservation of keratocyte viability is not an essential requirement for the primary function of these applications [21,22]. The primary limitations of cryopreservation, in contrast, are its technical complexity and high cost, which can hinder its application in resource-limited settings.

One pragmatic solution to these constraints is to co-locate the corneal lenticule biobank within an existing accredited tissue or cellular-therapy bank, thereby exploiting cryostorage, quality-management, donor-screening, and chain-of-custody infrastructure that is already in place. The first such service is OptiQ (Cordlife, Singapore), a private corneal lenticule banking service launched in 2021 under licence from the Singapore Ministry of Health. OptiQ is delivered through Cordlife’s Association for the Advancement of Blood and Biotherapies (AABB)- and FACT-accredited cord blood and tissue banking facility, which has been operating continuously since 2001. The viability and safety of this co-located banking workflow were validated in a preclinical study in which human KLEx-derived lenticules were collected at Singapore National Eye Centre (SNEC), transported to the Cordlife biobank, cryopreserved using a modified slow-cooling protocol for either 3 or 12 months, retrieved, and re-implanted into rabbit corneas. Both short- and long-term cryopreserved lenticules, although slightly less transparent than fresh controls owing to altered collagen-fibril packing, showed no evidence of rejection, cytotoxicity, haze, or neovascularisation during 16 weeks of in vivo follow-up [40]. The subsequent Ministry of Health licensing of OptiQ represents, to our knowledge, the first regulator-approved corneal lenticule banking service worldwide and provides a workable model for biobanks in other regions where dedicated stand-alone lenticule banking infrastructure is not yet economically justifiable.

Dehydration presents a simpler and more cost-effective alternative. There are various dehydration techniques, including air-drying under a laminar-flow hood, followed by storage in silica gel (as practiced by the Veneto Eye Bank Foundation, Venice, Italy) or controlled dehydration under low pressure and mild heat [41,42,43]. Studies have confirmed that these techniques effectively preserve the stromal architecture and collagen structure (Table 1). In addition, clinical studies have yielded promising results, demonstrating clear grafts without significant inflammation or rejection [44]. Nevertheless, further work is required to determine the maximum safe storage duration for dehydrated lenticules.

Hydrogel Nutrient Capsules are a relatively novel conservation approach that mimics the natural tear film environment using capsules composed of natural polysaccharides. Studies suggest that this method can maintain lenticule transparency, collagen organization, and cellular viability for up to one year (Table 1) [45,46]. A subsequent clinical study transplanting these capsule-preserved lenticules into ten patients reported no complications and good tissue transparency and integration [47].

A notable study by Xia et al. directly compared preservation in glycerol, silicone oil, and silica gel over four weeks. Results showed that while collagen arrangement remained regular in all groups, fibril density decreased. Notably, lenticules preserved in silica gel maintained transparency comparable to fresh controls, whereas storage in glycerol and silicone oil resulted in reduced transparency [48].

**Figure 3:**
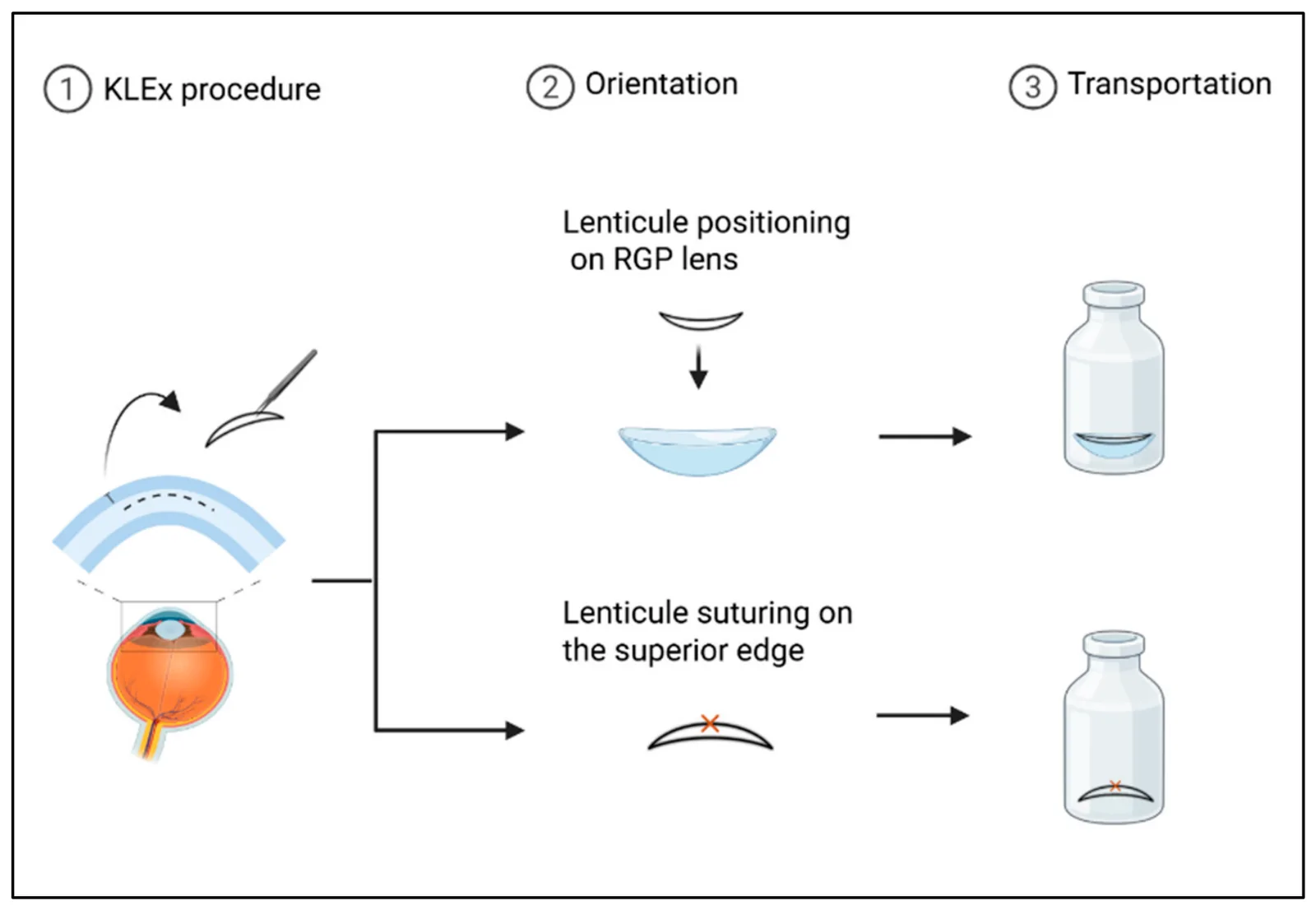
Preservation of the lenticule’s anatomical orientation can be achieved by either transferring the lenticule onto a marked rigid gas permeable (RGP) contact lens carrier or by suturing it to the superior edge at the time of collection. Figure created in BioRender. Barbaro, V. (2026) https://BioRender.com/hnjm2t8. (accessed on 1 September 2026).

A critical technical consideration is the preservation of the tissue’s native anatomical orientation. Lenticules extracted via KLEx procedures are aspheric, with distinct anterior and posterior surface curvatures designed to precisely alter corneal refraction. Furthermore, the collagen lamellae are more compact anteriorly and looser posteriorly. Implanting a lenticule in an inverted orientation could therefore result in suboptimal integration, irregular stromal healing, and unpredictable refractive outcomes due to the mismatch between the lenticule’s engineered shape and the host stromal bed. Therefore, marking the orientation during the initial extraction, storage, and transportation phases is crucial. Current best practices include either transferring the lenticule immediately onto a marked rigid gas permeable (RGP) contact lens carrier with the posterior surface facing down, ensuring its original anatomical orientation is preserved, or placing a nylon suture on the superior edge at the time of collection [24,36] (Figure 3). This is particularly important for toric lenticules, where rotational misalignment would nullify the intended correction. Therefore, further innovation in orientation marking and storage systems will be important to minimize errors and optimize clinical use of banked lenticules.

#### Quality Assurance, Screening, and Regulatory Considerations

Establishing a lenticule bank requires a comprehensive and well-defined framework for quality assurance, donor screening, traceability and regulatory compliance to ensure safety and efficacy. Although stromal lenticules are byproducts of refractive surgery, they must be managed with the same standards applied to any transplanted human tissue. Donor screening is essential and should include a detailed medical history and serological testing through Nucleic Acid Testing (NAT) to exclude transmissible infections such as HIV, hepatitis B/C, West Nile, and Human T-lymphotropic Viruses (HTLV). Quality assurance measures are also necessary to document key characteristics of each lenticule, including structural integrity, storage duration, and biometric parameters such as thickness, diameter, and refractive power. Each lenticule should be uniquely identified and traceable to the corresponding donor, with linkage to the relevant donor documentation, including serological results. This ensures traceability throughout processing, storage, and release for clinical application. In addition, predefined acceptance and rejection criteria should be established prior to clinical use. Lenticules should be considered suitable for clinical use only if they meet the applicable microbiological and storage requirements and exhibit complete structural integrity, with no evidence of fragmentation, tears, perforations, or other structural defects. Transparency and the absence of visible abnormalities should also be assessed. Biometric characteristics, including thickness and diameter, should be recorded in the bank’s database and used to support appropriate tissue allocation according to the intended clinical application and the surgeon’s requirements. Lenticules that do not meet the established quality, safety, or traceability requirements should be rejected and excluded from clinical use.

From a regulatory perspective, classification depends on the region. In the United States, KLEx lenticules are classified and regulated by the U.S Food and Drug Administration (FDA) as Human Cells, Tissues, and Cellular and Tissue-Based Products (HCT/Ps) under 21 CFR Part 1271. In the European Union, they are currently regulated under Directive 2004/23/EC and its implementing directives (2006/17/EC and 2006/86/EC), with oversight by national authorities [49,50,51]. Navigating the regulatory landscape remains complex, costly, and highly variable across regions, posing a significant challenge for broader clinical use and international distribution.

**Table 1:** Summary of preservation techniques of KLEx lenitucules. Techniques are summarized according to storage condition, preservation approach, structural integrity, keratocyte viability, and their main advantages and limitations.

Preservation Technique	Storage Condition	Approach	Structural Integrity	Keratocytes Viability	Advantages	Limitations
Cryopreservation	−80 to −196 °C	Cryopreservation with cryoprotectants (e.g., DMSO)	Alterations in collagen organization have been reported	Preserved	Well-established approach for long-term storage	Technically complex and costly
		Glycerol-based preservation	Maintained collagen integrity	Not preserved	Dehydrating and antimicrobial properties	Technically complex and costly
Dehydration	4 °C to 25 °C	Air-drying under a laminar-flow hood followed by silica gel storage	Stromal architecture and collagen organization maintained	Not preserved	Simple and cost effective	Limited evidence regarding long-term storage
		Controlled dehydration under low pressure and mild heat	Stromal architecture and collagen organization maintained	Not preserved	Simple and cost effective	Limited evidence regarding long-term storage
Hydrogel nutrient capsules	−80 °C	Encapsulation in a hydrogel composed of natural polysaccharides	Preserved stromal integrity and collagen organization	Preserved	Maintains transparency and collagen organization; fewer internal cavitation bubbles reported	Novel approach; limited evidence and clinical experience

## 3. Refractive Uses of KLEx Lenticules

During KLEx procedures, corneal curvature is altered in order to achieve refractive correction. In myopic correction, a stromal lenticule is extracted from the centre of the cornea, resulting in corneal flattening and a reduction in refractive power. On the other hand, in hyperopic correction, which requires an increase in corneal curvature, a steepening effect can be achieved by removing stromal tissue from the corneal periphery. In this context, femtosecond laser technology enables precise control of lenticule shape and thickness, allowing the creation of accurate refractive lenticules [17,18].

### 3.1. KLEx Lenticule Implantation for Hyperopia Correction

Hyperopia remains one of the most common refractive errors worldwide, with pooled prevalence estimates of 36% in Asia, 49% in America, 40% in Europe, and 47% in Africa [52,53]. Its frequency increases with age, and moderate to high hyperopia remains a significant cause of visual impairment and dependence on corrective eyewear. Although spectacles and contact lenses offer effective compensation, many patients seek surgical correction for greater independence from glasses. Currently, excimer laser-based procedures such as photorefractive keratectomy (PRK) and laser-assisted in situ keratomileusis (LASIK) are the most widely accepted surgical options for hyperopia correction [54]. However, these laser-based approaches require peripheral stromal ablation for central corneal steepening, a biomechanically challenging process that induces stromal thinning and may compromise corneal stability [55,56]. Compared with myopic correction, hyperopic treatments are generally less predictable and more prone to regression [57,58,59]. In addition, higher-order aberrations and loss of best-corrected visual acuity (BCVA) have been associated with these procedures, particularly in higher degrees of hyperopia [60]. In this context, KLEx-derived lenticule implantation has emerged as a promising alternative, since, unlike ablative procedures, this approach adds stromal tissue rather than removing it, thereby avoiding stromal loss and better preserving corneal biomechanics.

Based on Barraquer’s law of thickness, in order to steepen the anterior corneal curvature, tissue is added to the centre of the cornea or removed from the corneal periphery. Essentially, hyperopia correction is achieved when corneal curvature is increased, and consequently, its refractive power [61]. However, as mentioned earlier, early stromal keratophakia resulted in no significant improvement in visual outcome and various postoperative complications that were partially due to the technically complex and expensive equipment necessary for performing the procedure [11]. Today’s advancement of femtosecond technology has enabled us to overcome the issues related to the cutting accuracy of the microkeratome and has provided us with precise refractive lenticules.

The first clinical application of femtosecond laser-assisted keratophakia was reported by Pradhan et al., who implanted in a lamellar pocket at 110 μm an allogeneic KLEx lenticule with a central thickness of 127 μm obtained from a myopic donor for correction of high hyperopia (+12.00 −1.50 × 155) (Table 2). One year postoperatively, retinoscopy refraction was +7.50 −3.00 × 150, reflecting a notable undercorrection. This was accompanied by posterior surface elevation and a central anterior chamber bulge, which likely contributed to the limited refractive effect. This report highlighted the biomechanical challenges encountered in this early application of lenticule addition [25].

Shortly thereafter, Ganesh et al. evaluated the feasibility of using cryopreserved Small-Incision Lenticule Extraction (SMILE)-derived lenticules for hyperopic correction. In a stromal pocket at a depth of 160 μm, the authors implanted a cryopreserved lenticule in nine eyes with moderate hyperopia or aphakia (Mean SE of +4.50 ± 1.1 D). All eyes achieved postoperative uncorrected distance visual acuity (UDVA) equal to or better than preoperative corrected distance visual acuity (CDVA). Mean residual spherical equivalent (SE) in hyperopic eyes was +0.6 D, whereas the aphakic case remained substantially undercorrected (+4.1 D). Central keratometry increased by 3.5 D in the central 3 mm zone, with a hyperprolate shift in Q value (−0.38 to −0.89). The authors attributed the substantial undercorrection in the aphakic case to failure to compensate for back vertex distance (the difference between spectacle plane and corneal plane refractive power) [36]. Nonetheless, these preliminary results suggested that femtosecond laser-assisted stromal keratophakia for hyperopia correction may offer improved stability and reduced regression compared with hyperopic LASIK.

Recently, the same group published a 5-year follow-up of 42 eyes. Their results confirmed long-term stability and a significant mean SE reduction from +5.50 ± 1.96 to +0.66 ± 1.17 D [62]. These findings are comparable with the long-term (5 years) findings of hyperopic LASIK that showed a reduction from +3.74 to +0.84 D reported by Dave et al. [63]. In total, 30 eyes (71%) were within ±1.00 D of SE correction, and efficacy and safety indices were 0.86 ± 0.19 and 1.17 ± 0.39, respectively. In addition, stability at 2 weeks postoperatively was 0.64 ± 1.05 D, which showed a non-significant increase to 0.66 ± 1.17 D at the last postoperative visit, indicating early and lasting stability. Nonetheless, enhancement (four eyes) and lenticule explantation due to suspected rejection (four eyes) were reported. In this context, the use of electron-beamed tissue could mitigate such complications by improving tissue stability and reducing immunogenicity [64].

Sun et al. reported autologous lenticule implantation in five patients with unilateral myopia and contralateral hyperopia. In this approach, a lenticule extracted from the myopic eye was implanted under a 110 μm flap in the hyperopic eye, preceded by stromal excimer ablation to correct residual refractive error. At one year postoperatively, lenticule position remained stable, and mean hyperopic reduction was approximately 5.5 D. Even though visual acuity improved in most cases, residual refractive error varied widely, from +1.13 to −2.63 D at the last follow-up visit, emphasizing limited predictability [65]. In a subsequent prospective study of 10 patients, the same group proposed a predictive model to estimate achieved correction based on lenticule refractive power (LAC = 1.224 × LRP − 0.063; R^2^ = 0.92). In total, 6 of 10 eyes (60%) achieved a postoperative refraction within ±1.00 D of the intended target, and no loss of CDVA was observed. This represented an initial attempt to standardize lenticule selection and improve refractive planning for lenticule implantation [66].

Lin et al. evaluated visual outcomes and corneal densitometry (CD) of 22 eyes after autologous (*n* = 8) and allogeneic (*n* = 14) lenticule intrastromal keratoplasty for correction of moderate to high hyperopia. Lenticules were implanted under a 110 μm flap. At 6 months postoperatively, 5 of the 14 treated eyes in the allogeneic group and 6 of the 8 eyes in the autologous group achieved postoperative UDVA equal to or better than the preoperative CDVA. Consistent with previous reports, no eye lost more than one line of CDVA, and 50% of all eyes achieved SE within ±1.00 D of the targeted correction. Additionally, CD values of the anterior layer in the autologous group decreased to preoperative values 1 month postoperatively. Conversely, in the allogeneic group, CD values of the anterior and central layers remained significantly higher than the preoperative values at 6 months postoperatively. This difference is likely attributed to the better biocompatibility and reduced stromal immunological rejection of the autologous lenticule [67]. These findings are in agreement with Hou et al., who reported UDVA equal to or better than the preoperative CDVA at 6 months following allogeneic small-incision intrastromal lenticule implantation (SILI). Furthermore, results showed a significant increase in CD values from 16.60 ± 1.89 preoperatively to 18.19 ± 2.68 at 6 months post-SILI (*p* < 0.001), particularly in the anterior and mid stromal layer within the surgical zone [68].

In a subsequent comparison study, Lin et al. evaluated the long-term visual outcome and higher-order aberrations (HOAs) following FS-LASIK and small-incision lenticule intrastromal keratoplasty (SMI-LIKE) for moderate to high hyperopia correction in a total of 30 eyes (FS-LASIK, *n* = 20; SMI-LIKE, *n* = 10). Results demonstrated a higher correlation coefficient of attempted versus achieved SE in the SMI-LIKE group compared to FS-LASIK (0.89 and 0.69, respectively). Mean postoperative SE was 4.81 ± 1.19 D in the SMI-LIKE group and 5.61 ± 1.08 D in the FS-LASIK group; however, the difference was not statistically significant. Importantly, total HOAs and coma significantly increased in both groups (*p* > 0.01). In contrast, spherical aberration (SA) significantly decreased following both procedures, with SMI-LIKE inducing smaller negative SA changes than FS-LASIK. The authors attributed this difference to the larger optical zone and more natural transition zone in the SMI-LIKE group, as well as the greater observed Q value changes in the FS-LASIK group. In this context, Wu et al. recently reported visual and refractive outcomes following femtosecond laser-assisted lenticule intrastromal keratoplasty in moderate to high hyperopia correction. In total, 61% of all eyes achieved UDVA better than or equal to preoperative CDVA, while 78% had SE within ± 1.00 D from the attempted correction. Nonetheless, despite these favourable outcomes, a significant increase in vertical coma and SA was observed [69]. Overall, this study highlights the challenges associated with hyperopic correction, as despite improvement in SE, induced HOAs remain a significant concern [70]. Similarly, Zhang et al. also compared visual acuity and refractive outcome following FS-LASIK (*n* = 22) and SMI-LIKE (*n* = 20). Results showed an improvement in uncorrected near visual acuity (UNVA) and UDVA in both groups 1 year postoperatively. Consistent with previously reported results, UDVA was equal to or better than the preoperative CDVA in 16 eyes (80.0%) in the SMI-LIKE group and 8 eyes (36.4%) in the FS-LASIK group. Moreover, the amount of postoperative residual hyperopia in the SMI-LIKE group was significantly lower than in the FS-LASIK group, as nine eyes (45%) of the SMI-LIKE group and three eyes (13.6%) of the FS-LASIK group achieved an SE within ±0.50 D of the intended correction [71].

Flap-based techniques were also associated with more potential complications. In a single-patient case report, Moshirfar et al. reported epithelial ingrowth and flap necrosis following implantation beneath a 100 μm deep femtosecond LASIK flap in a highly hyperopic eye (+6.00 −1.00 × 40). Although refractive improvement was achieved (−1.25 × 71 D), this case reinforced the inherent limitations of flap-based approaches and supported the shift toward small-incision or pocket techniques [72].

An additional variation in the use of lenticule implantation for hyperopic correction has been described in the context of iatrogenic refractive error following LASIK. In a case study reported by Lazaridis et al., severe post-LASIK hyperopia combined with high astigmatism and marked stromal thinning (+6.50 −9.00 × 84) was treated by implanting a toric myopic lenticule (−4.00 −4.25 × 14) beneath the pre-existing LASIK flap to restore both corneal volume and refractive power. The procedure resulted in a substantial reduction in astigmatism but a stronger myopization (−6.50 −4.00 × 70). Subsequently, after implanting a toric myopic implantable collamer lens, the patient regained UDVA [73]. This case highlights the adaptability of lenticule transplantation potential as a reversible and tissue-conserving option when conventional approaches are limited.

Subsequent studies focused on small-incision intrastromal implantation. Liu et al. evaluated 14 eyes undergoing allogeneic lenticule implantation in a 100 µm pocket for moderate to high hyperopia (+3 to +8 D). At 2 years, 78.6% of eyes maintained CDVA, and mild overcorrection was observed (mean SE from +5.53 D to −0.60 D). Notably, no significant changes in anterior or posterior keratometry were detected during follow-up, suggesting good structural stability [74]. In a follow-up study, the same group compared the changes in posterior corneal surface following small-incision lenticule intrastromal keratoplasty (SMI-LIKE) and femtosecond laser-assisted lenticule intrastromal keratoplasty (FS-LIKE). SMI-LIKE induced transient posterior corneal surface changes that returned to baseline during follow-up, whereas FS-LIKE exhibited a different pattern of posterior surface elevation changes. The authors attributed these differences to weakening of the anterior surface and reduced biomechanical support associated with flap creation compared with pocket-based implantation [75].

Despite favourable safety outcomes, refractive predictability remains influenced by multiple surgical and biological factors, including lenticule thickness, lenticule implantation depth, and corneal epithelium remodelling. Damgaard et al. conducted an ex vivo study where they demonstrated that shallower implantation (100 µm) achieved approximately 78% of attempted correction compared with 50% at 160 µm [76]. These findings suggest that anterior placement maximizes anterior curvature changes, whereas deeper placement may reduce refractive efficiency, possibly through posterior curvature compensation. These data indicate that the ideal depth at which the lenticule must be implanted for accurate results and long-term stability after tissue addition for hyperopia is debatable. Based on available evidence, implantation depth between 120 and 130 µm may represent a theoretical balance between maximizing anterior curvature effect and minimizing posterior surface alteration. However, further comparative clinical studies are required. In addition, lenticule power must be adjusted according to implantation depth to optimize predictability.

Epithelial remodelling, like corneal biomechanical changes following tissue implantation, represents another important factor influencing long-term refractive stability. Recently, Dong et al. investigated epithelial thickness changes following small-incision lenticule intrastromal keratoplasty for hyperopia. A donut-shaped epithelial remodelling pattern was observed, similar to a post-LASIK profile. The thinnest epithelium was 48.18 ± 2.86 μm, and the thickest was 58.06 ± 5.56 μm, which is consistent with epithelial thickness distribution in a normal cornea. A significant correlation was found between implanted lenticule refractive power and central epithelial thinning, suggesting that epithelial compensation may partially modulate achieved refractive outcomes. As for the visual outcome, a total of 10 eyes (59%) had postoperative UDVA equal to or better than preoperative CDVA, and a total of 71% of eyes were within ±1 D. However, lower degrees of hyperopia tended to be overcorrected, while higher degrees tended to be undercorrected [77]. These findings suggest that epithelial remodelling may partially compensate for stromal curvature changes and could contribute to the refractive variability observed in hyperopic lenticule implantation.

A distinct strategy avoids the use of SMILE-derived tissue altogether and instead employs an excimer laser-shaped allograft corneal inlay (ACI) prepared from donor corneal stroma by a certified eye bank. Tanriverdi et al. evaluated this approach in a prospective series of 28 hyperopic eyes of 16 patients, implanting a 6.0 mm ACI (Allotex TransForm), whose refractive power was customized to each eye, beneath a 110 μm femtosecond LASIK flap [78]. At 12 months, the mean manifest refraction spherical equivalent (MRSE) decreased from 3.60 ± 1.51 D to 0.21 ± 0.56 D (*p* < 0.001), with 89% and 57% of eyes within ±1.00 D and ±0.50 D of the intended correction, respectively. UDVA and UNVA improved from 0.33 ± 0.22 and 0.17 ± 0.13 to 0.75 ± 0.22 and 0.72 ± 0.19 (Snellen decimal), while CDVA remained unchanged, and the mean keratometry and central corneal thickness increased from 42.57 ± 0.81 D and 557.5 ± 43.0 μm to 44.8 ± 1.4 D and 597.1 ± 58.1 μm, respectively. A regression of approximately 0.44 ± 0.59 D was observed between the 6- and 12-month visits, and no diffuse lamellar keratitis, epithelial ingrowth, haze, rejection, or decentration was recorded. Notably, the predictability achieved with the individually power-adjusted ACI compared favourably with that reported for hyperopic LASIK and for lenticules harvested from myopic donors, underscoring the value of pre-shaping the graft to the recipient’s refractive error rather than relying on a lenticule of fixed myopic power.

Overall, KLEx-derived lenticule implantation represents a minimally invasive, tissue-additive strategy for hyperopia correction. While safety and long-term stability appear favourable, refractive predictability remains influenced by implantation depth, lenticule power selection, optical zone size, and epithelial remodelling. Further refinement of surgical planning and standardized nomograms may improve accuracy and reduce variability.

**Table 2:** Clinical outcome of KLEx lenticule implantation for hyperopia correction. Studies are summarized according to the number of eyes treated, lenticule type (allogeneic refers to a lenticule obtained from another individual, whereas autologous refers to a lenticule obtained from the recipient’s contralateral eye), technique/depth, and preoperative and postoperative refractive outcome (Sphere, cylinder, and SE). ^°^ Case report; ^#^ case series; ^Δ^ clinical study; * retrospective study.

Study	Eyes (n)	Lenticule Type	Technique/Depth	Sphere (D)		Cylinder (D)		SE (D)	
				Pre-op	Post-op	Pre-op	Post-op	Pre-op	Post-op
^°^ Pradhan et al. (2013) [25]	1	Allogeneic	Pocket (110 µm)	12.00	−1.50	7.50	−3.00	15.75	−3.00
^#^ Ganesh et al. (2014) [36]	9	Allogeneic	Pocket (160 µm)	5.11 ± 2.37	0.61 ± 1.10	0.28 ± 0.36	0.78 ± 0.38	4.5 ± 1.11	1.14 ± 1.14
* Brar and Ganesh et al. (2022) [62]	42	Allogeneic	Pocket (160 µm)	5.24 ± 1.96	0.56 ± 0.94	0.51 ± 0.48	0.19 ± 0.67	5.54 ± 1.96	0.66 ± 1.18
^#^ Sun et al. (2015) [65]	5	Autologous	Flap (110 µm) preceded by excimer ablation	4.75 ± 2.27	−0.85 ± 1.43	−0.8 ± 0.71	−0.65 ± 0.45	4.34 ± 1.93	−1.62 ±0.78
^Δ^ Li et al. (2017) [66]	10	Autologous	Flap-based	5.20 ± 1.77	−0.18 ± 1.39	−1.48 ± 1.68	−0.70 ± 0.42	4.46 ± 1.97	−0.53 ± 1.45
^Δ^ Lin et al. (2023) [67]	22	Allogeneic (*n* = 14)	Flap-based (110 µm)	6.55 ± 2.45	NA	−1.05 ± 0.8	NA	6.03 ± 2.54	−0.24 ± 0.96
		Autologus (*n* = 8)		4.59 ± 1.36	NA	−1.22 ± 1.78	NA	3.98 ± 1.87	−0.61 ± 1.60
* Hou et al. (2022) [68]	31	Allogeneic	Pocket (120 µm)	NA	NA	NA	NA	5.95 ± 2.1	0.52 ± 0.82
^Δ^ Lin et al. (2023) [70]	10	Allogeneic	Pocket (100 µm)	6.10 ± 1.05	NA	−0.98 ± 0.34	NA	5.61 ± 1.08	−0.60 ± 1.20
^Δ^ Zhang et al. (2022) [71]	42	Allogeneic	Pocket (120 µm; n = 20)	4.62 ± 0.97	0.96 ± 0.37	0.68 ± 0.53	−0.66 ± 0.48	4.96 ± 0.93	0.63 ± 0.28
			Flap (110 µm; n = 22)	4.66 ± 0.76	1.22 ± 0.61	0.95 ± 0.58	−0.41 ± 0.46	5.13 ± 0.70	1.01 ± 0.57
^°^ Moshirfar et al. (2020) [72]	1	Allogeneic	Flap-based	6.50	0.00	−1.00	−1.25	6.00	−0.62
^°^ Lazaridis et al. (2016) [73]	1	Allogeneic	Flap-based (post-LASIK)	6.50	−6.25	−9.00	−4.00	2.00	−8.25
^#^ Liu et al. (2021) [74]	14	Allogeneic	Pocket (100 µm)	5.99 ± 1.46	0.48 ± 1.13	−0.89 ± 0.33	−1.59 ± 0.95	5.54 ± 1.45	−0.60 ± 1.20
* Dong et al. (2024) [77]	17	Allogeneic	SMI-LIKE (small-incision)	NA	NA	−1.26 ± 0.95	NA	5.37 ± 1.80	NA
^#^ Wu et al. (2025) [69]	18	Allogenic	Flap (110–120 µm)	6.64 ± 0.99	NA	−0.97 ± 0.54	NA	6.15 ± 0.97	−0.01 ± 0.96
^#^ Tanriverdi et al. (2021) [78]	28	Allogeneic (excimer-shaped ACI)	Flap (110 µm)	NA	NA	NA	NA	3.60 ± 1.51	0.21 ± 0.56

### 3.2. KLEx Lenticule Implantation for Presbyopia Treatment

Presbyopia is the most prevalent refractive error, with an estimated 2.1 billion cases by 2030 due to global population ageing [79]. It is characterized by a progressive decline in the crystalline lens’ ability to accommodate, resulting in reduced ability to focus on near objects. Symptoms typically begin around the age of 40 and worsen with age. In most cases, presbyopia is managed by wearing spectacles with convex lenses. However, traditional glasses provide clear vision only at a fixed focal distance and therefore need to be removed for distance vision [80]. While bifocal glasses could offer a solution to this limitation, they often cause image jump or distortion of the peripheral image. In addition, there is a growing desire for spectacle independence. In this context, common approaches for presbyopia correction include multifocal and accommodating intrastromal lenses (IOLs), corneal presbyopic laser procedures, and corneal inlay implantation, which have emerged as potential therapeutic strategies [81].

The first inlays were introduced by Jose Barraquer in 1949. They were composed of polymethylmethacrylate or glass, and despite initial clinical signs of success, high rates of implant extrusion and corneal necrosis from reactions to the material were reported, leading to their abandonment [61]. Today, the discovery of more biocompatible materials, like hydrogels, and the ability of the femtosecond laser to make very precise intrastromal tunnels to implant the lenticules accurately, have revived the interest in this approach [82].

Several synthetic inlays with different principles and modes of action have been developed and commercialized. Among them, the Kamra small-aperture inlay (Acufocus Inc., Irvine, CA, USA) is designed to increase the depth of focus by using the pinhole principle to improve near vision. The Presbia Flexivue Microlens (Presbia Cooperatief U.A., Amsterdam, The Netherlands), a hydrophilic refractive inlay engineered to provide multifocality, and Raindrop^TM^ (Revision Optics Inc., Lake Forest, CA, USA), which reshaped the anterior curvature but was ultimately discontinued due to postoperative complications [11,83]. Overall, synthetic inlays have been associated with stromal inflammatory response, including thinning, melting, opacification, and decentration, likely due to impaired nutrient flow across the inlay [84,85].

In this context, myopic KLEx lenticules have emerged as a promising biological alternative to synthetic inlays. As allogeneic tissue, they offer superior biocompatibility, preserve biological nutrient flow, and reduce the risk of stromal inflammatory response and extrusion [86]. Functionally, the mechanism is similar to the Raindrop inlay, as the refractive power change is caused by the mechanical rise in the anterior stroma [87,88].

In a proof-of-concept study in a non-human primate model performed by Liu et al., SMILE-derived lenticules (with a central thickness of 65 μm) were trephined into a 3 mm diameter and implanted into a stromal pocket at a depth of 120 µm, in an autogenic or xenogeneic manner. At 6 months postoperatively, implanted lenticules, regardless of autogenic or xenogeneic, were well centred and integrated with the surrounding stroma. Simulated keratometric (Sim K) value increased by 1.08–2.3 D, central corneal height increased by 7.7–9.3 μm, and the asphericity Q values changed by −0.25 to −0.36. It is important to note that total corneal thickness increased by 29–36 μm despite implanting a 65 μm lenticule. The authors explained that this was attributed to postoperative epithelial remodelling after a refractive procedure, which also contributed to the anterior elevation measuring 1.5–1.8 times the lenticule physical diameter [89].

Jacob et al. implanted centrally trephined 1 mm SMILE-derived lenticules of specified thickness (61.5 ± 3.32 µm) under a femtosecond laser-created cap of 120 µm depth in four patients. Postoperatively, corneal topography demonstrated increased hyperprolateness in the central 3 mm zone. This was accompanied by an improved UNVA between three and five lines without any change in UDVA. At 6 months postoperatively, all lenticules remained centred, and UDVA was maintained, with no report of dysphotopsia or troublesome night glare or halos [86].

In January 2026, the FDA granted a conditional Investigational Device Exemption (IDE) for the Allotex ALLO-1 corneal allograft (the next-generation TransForm-family product) for presbyopia, authorizing the initiation of a US clinical study. The original TransForm corneal allograft (TCA) has had commercial market clearance in Europe and selected other regions since 2019–2020. This allogeneic inlay is a decellularized corneal graft, measuring 2.6 mm in diameter and 20–22 μm in thickness, cut from a donor cornea using an excimer laser and sterilized with electron beam radiation. It is implanted under a femtosecond laser-created intrastromal flap (approximately 100–110 μm thick). In their recently published long-term multicentre study, 101 patients with emmetropic presbyopia were treated, with follow-up data available for 94 at 6 months and 71 at 4 years. The authors reported significant and stable improvement in near and intermediate vision while maintaining distance vision. At 6 months postoperatively, mean binocular UDVA, intermediate (UIVA), and UNVA were −0.11 ± 0.07, 0.09 ± 0.18, and 0.03 ± 0.11 logMAR, respectively. Notably, 98.9% of patients achieved a UNVA of 20/40 or better at 6 months, which was maintained at 98.6% at 4 years compared to 16% preoperatively. Nonetheless, the inlay was explanted in 3 eyes before 6 months due to anisometropia intolerance, with no long-term sequelae [28].

Despite these promising results, clinical evidence supporting the use of allogeneic inlays for the management of presbyopia remains relatively limited, and further studies are required to attest to the efficacy and safety.

## 4. Therapeutic Uses of KLEx Lenticules

### 4.1. The Use of KLEx Lenticule for the Treatment of Keratoconus and Post-LASIK Ectasia

**Figure 4:**
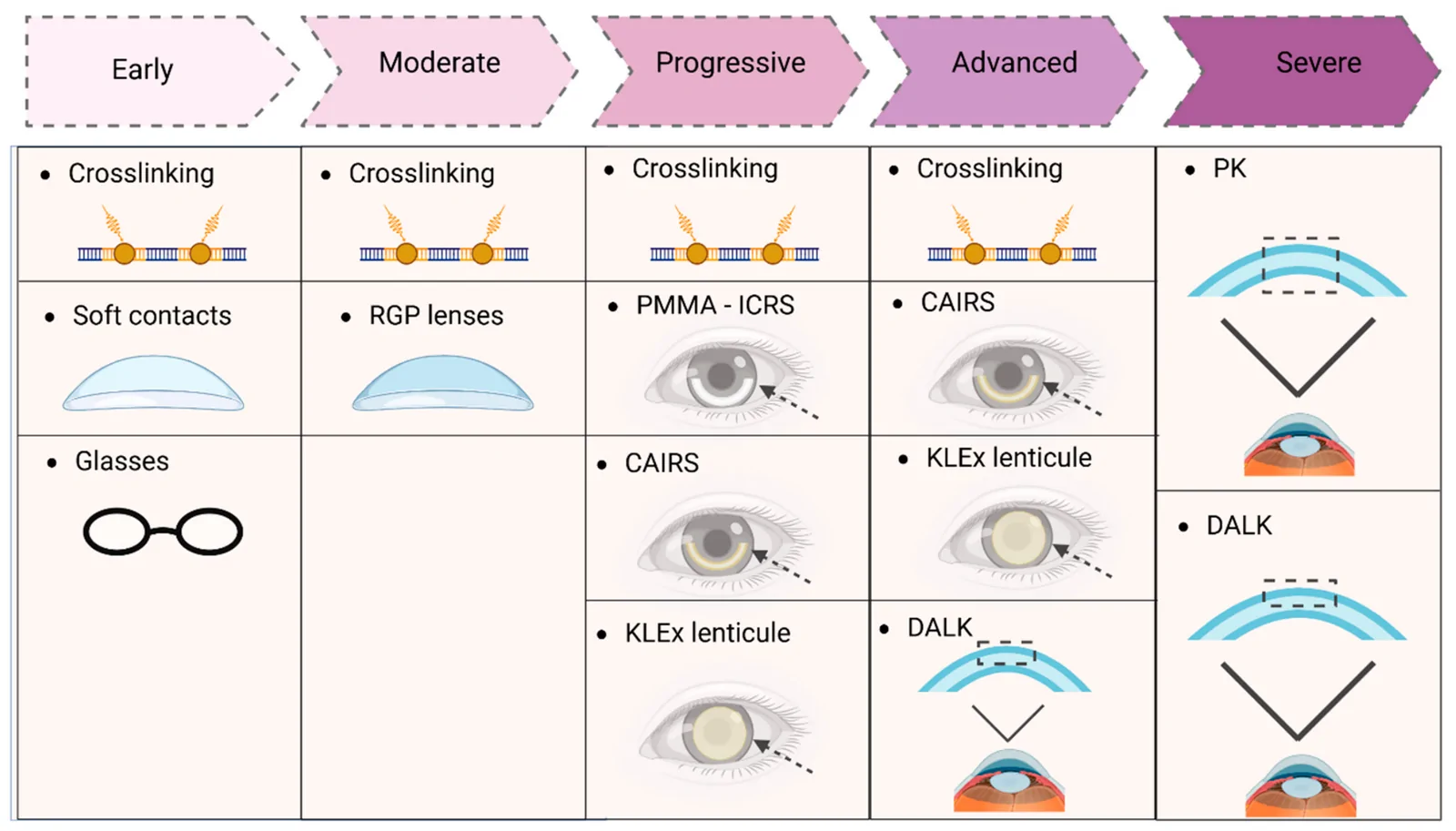
Schematic representation of management strategies for keratoconus across disease severity. Corneal crosslinking (CXL) is indicated from the early to advanced stages to halt disease progression. In early stages, correction is achieved with glasses or contact lenses, while moderate or progressive cases can require the use of rigid gas permeable (RGP) contact lenses or intrastromal corneal ring segments (ICRS), either synthetic polymethyl methacrylate (PMMA) or allogeneic, such as CAIRS. KLEx lenticle implantation represents an emerging stromal augmentation strategy for progressive and advanced cases. In advanced or severe cases, surgical intervention through deep anterior lamellar keratoplasty (DALK) or penetrating keratoplasty (PK) becomes necessary. Figure created in BioRender. Barbaro, V. (2026), https://BioRender.com/k8nbmbk. (accessed on 1 September 2026).

Ectatic disorders such as keratoconus or post-LASIK ectasia are characterized by progressive steepening and thinning of the cornea, leading to visual impairments and potential corneal blindness. The current treatments available depend on the severity of the disease. In early stages, correction is managed with glasses or contact lenses, while moderate or progressive cases can require the use of rigid gas permeable (RGP) contact lenses or intrastromal corneal ring segments (ICRS), either synthetic, made from polymethyl methacrylate (PMMA), or allogeneic, such as CAIRS. A common treatment for halting progression is collagen cross-linking (CXL), which uses riboflavin and ultraviolet-A (UV-A) in order to provide biomechanical stability and slow corneal thinning. However, CXL does not improve corneal refraction. In severe or advanced cases, where the cornea is too thin for CXL (>400 µm), surgical intervention through deep anterior lamellar keratoplasty (DALK) or penetrating keratoplasty (PK) becomes necessary (Figure 4). It is important to mention that while beneficial, these treatments have their limitations. RGP lenses are often poorly tolerated by many patients, PMMA ICRS can cause complications such as stromal melting and inflammation due to poor biointegration, and corneal transplantation carries the risks of graft rejection and limited donor availability [90,91,92]. It is here that implantation of KLEx lenticules for corneal thickness restoration and visual acuity improvement could be a compelling alternative. Compared to traditional keratoplasty, KLEx lenticule implantation is simpler and carries a significantly lower risk of graft rejection. This is because the lenticule is a smaller graft that is strategically implanted within the stroma, away from the limbal lymphatic vessels and shielded from immune cells in the tear film and aqueous humour. Mechanistically, it provides volume augmentation and corneal reshaping and flattening, leading to regularization and pachymetric restoration.

Early animal studies validated the technical feasibility and safety of lenticule addition as a volumetric corneal augmentation strategy [23,24]. The primary goals were to increase stromal thickness, improve optical regularity, and enable crosslinking in corneas previously too thin for treatment.

The first clinical report of this approach was by Sachdev et al. They placed a fresh stromal lenticule obtained from the SMILE procedure over the thinnest area of the cone in three patients (after de-epithelialization) and proceeded with the CXL procedure. No intraoperative or postoperative complications were noted, and complete epithelium remodelling occurred within 3 to 5 days. An even demarcation line indicative of CXL was visible on corneal AS-OCT in all cases, and histopathologic analysis of the refractive lenticule revealed increased collagen rigidity and compactness, confirming the effects of crosslinking [93].

Subsequently, Ganesh and Brar et al. evaluated femtosecond intrastromal lenticule implantation (FILI) combined with accelerated collagen cross-linking in six eyes with progressive central keratoconus, using cryopreserved lenticules that were centrally punched to 3 mm to create a donut-shaped graft. The authors reported that using elevation topography to calculate the required amount of tissue to be added proved unreliable due to surface irregularity and variability of the best-fit sphere in the ectatic area. Therefore, for simplicity, lenticule selection was instead based on the recipient’s SE refraction. After 6 months, significant improvements were observed in UDVA and CDVA (from 1.06 ± 0.48 to 0.38 ± 0.27 logMAR and from 0.51 ± 0.20 to 0.20 ± 0.24 logMAR, respectively), and in manifest SE (−3.47 ± 1.15 to −1.77 ± 1.7 D). Mean keratometry flattened by 3.42 ± 2.09 D (3 mm zone) and 1.70 ± 1.31 D (5 mm zone), while central and midperipheral pachymetry increased by 18.3 ± 7.3 μm and 33.0 ± 8.8 μm, respectively. Higher-order aberrations decreased, and no adverse events occurred, supporting the feasibility and safety of FILI with accelerated CXL in low to moderate keratoconus [26].

Building on these advances, Dong et al. further validated the combined use of femtosecond intrastromal lenticule implantation (FILI) and accelerated collagen cross-linking (CXL) in a prospective series of nine eyes from eight patients with advanced keratoconus. A convex stromal lenticule obtained via SMILE was inserted through a 4 mm intrastromal pocket incision, followed by CXL. At 6 months postoperatively, LogMAR UDVA (1.04 ± 0.21) did not differ significantly from preoperative values (1.13 ± 0.35, *p* = 0.268). Six eyes achieved an average LogMAR BCVA of 0.08 ± 0.07 with RGPs, while patients unable to tolerate RGPs showed no visual improvement. In accordance with previous studies, corneal biomechanical properties improved significantly. SP-A1 increased from 36.17 ± 4.70 to 53.47 ± 3.55, ARTh from 129.19 ± 8.63 to 320.61 ± 54.47, and the integrated radius decreased from 17.18 ± 3.26 to 13.36 ± 2.64. The mean central anterior keratometry (Km) increased from 56.99 ± 2.17 D to 58.31 D at 6 months, though this change was not statistically significant (*p* = 0.139). Importantly, corneal thickness at the thinnest point increased significantly from 392.67 ± 6.46 μm to 482.67 ± 12.08 μm postoperatively, with no further significant changes during follow-up, and central corneal epithelial thickness (0–1 mm zone) also increased from 44.78 ± 4.06 μm to 49.78 ± 4.09 μm at 6 months [94].

In order to better understand the effect of convex lenticule implantation on the corneal surface, Sun et al. recently examined changes in the anterior and posterior corneal surfaces after stromal lenticule addition keratoplasty (SLAK) combined with CXL in 20 eyes with severe keratoconus over a 3-month follow-up period. They found that the anterior corneal curvature (K1 and K2) at 3 mm, 5 mm, and 7 mm zones increased significantly 1 month postoperatively (*p* < 0.05) and remained stable throughout the follow-up period. The authors interpreted this early steepening as “pseudoprogression” induced by the lenticule convex profile. This was further supported by the absence of progressive posterior corneal changes. Posterior K2 readings at 5 mm and 7 mm showed that the posterior elevation consistently remained stable, with only limited flattening observed centrally. These results indicated that the morphological changes occurred mainly in the front corneal surface related to keratometry and elevation values.

Since convex lenticules can induce anterior steepening, Mastropasqua et al. proposed implantation of negative meniscus-shaped stromal lenticules. In a study involving 10 patients with stage III–IV keratoconus, they implanted a hyperopic lenticule into intrastromal pockets, created by femtosecond laser flap-cut. These lenticules had a 6 mm optical zone and a 0.7 mm transition zone in the periphery. The central minimal lenticular thickness was 30 μm, and the peripheral maximal thickness was 148 μm. After 6 months, both UDVA and CDVA significantly improved (from 1.58 ± 0.36 to 1.22 ± 0.37 logMAR and from 1.07 ± 0.17 to 0.70 ± 0.23 logMAR, respectively). Corneal topography revealed a reduction in anterior mean curvature from 58.69 D preoperatively to 53.59 D at 6 months postoperatively. Q values also decreased significantly, while AS-OCT demonstrated increased central and midperipheral corneal thickness in correspondence with the values programmed during lenticule preparation [95]. More recently, Sinha et al. demonstrated comparable results in their prospective study where they evaluated the outcomes of intrastromal negative meniscus lenticule implantation followed by accelerated collagen crosslinking in 10 eyes with progressive keratoconus. At 12 months postoperatively, mean UDVA improved from 1.43 ± 0.39 to 1.32 ± 0.31 logMAR. Corneal flattening was observed across keratometry all measurements, with K1 decreasing from 58.44 ± 5.59 to 55.57 ± 5.68 D, K2 from 63.35 ± 6.86 to 59.35 ± 7.1, and Kmax from 71.07 ± 7.95 to 66.29 ± 7.54 D. Astigmatism also decreased from 4.89 ± 3.35 to 3.88 ± 2.35 D. Mean thinnest pachymetry increased significantly from 354.9 ± 28.52 to 462.5 ± 36.30 μm (*p* = 0.0001). Additionally, HOA decreased from 3.45 ± 3.41 to 2.12 ± 1.43, while mean SE decreased from −10.95 ± 4.7 D to −7.53 ± 2.96 D at 12 months [96].

Epithelial thinning at the corneal apex surrounded by epithelial thickening is a characteristic feature of keratoconic cornea [97]. Therefore, in addition to stromal remodelling, epithelial remodelling is also relevant in this context. Based on these observations, in a prospective study, Nubile et al. evaluated changes in the corneal epithelial and stromal thickness following femtosecond laser-assisted stromal lenticule addition keratoplasty (SLAK) in patients with advanced keratoconus. Comparable with the previous study, at 6 months postoperatively, the average anterior Sim-K decreased from 59.63 ± 7.58 to 57.19 ± 6.33 D. Mean corneal thickness increased by 64 ± 25 μm, while the corneal thinnest point increased by 73 ± 27 μm despite the implantation of lenticules with a central thickness of 30 μm. The authors attributed this finding to remodelling of the recipient stromal bed and the exclusion of lenticule interface boundaries in the OCT measurements. In addition, epithelial thickness in the central area stabilized 1 month postoperatively, with an average of 7 ± 3 μm, suggesting an early remodelling plateau. Similar to the epithelial remodelling pattern observed after a laser refractive procedure for corneal flattening, in this study, the flattening effect was associated with significant epithelial thickening at the cone apex. Moreover, epithelial remodelling also occurred in both the inner annular (mid-peripheral) and outer annular (peripheral) regions, with a decrease in the inner area of 4 ± 2 μm and a significant increase in the outer area of 14 ± 5 μm [98]. Collectively, these findings indicate that negative meniscus lenticule configuration effectively flattened the ectatic cone and augmented stromal volume, further supporting the clinical efficacy of femtosecond laser-assisted SLAK as a treatment option for advanced keratoconus.

Pradhan et al. reported an alternative sutureless femtosecond laser-assisted intrastromal lamellar keratoplasty in a young patient with severe keratoconus. A 400 μm thick lenticule was prepared by performing DALK in donor corneal tissue, followed by excimer laser ablation to remove 50 μm of tissue anteriorly. The lenticule was inserted through a 3 mm incision into the recipient cornea without sutures. At one-year follow-up, UCVA improved from −5.00–3.50 × 170 to −2.50–3.50 × 125, and *k*_max_ decreased from 64.08 D to 56.74 D, demonstrating effective flattening of the ectatic cornea. This report offers an alternative approach for lenticule implantation in keratoconus, reducing surgical trauma and providing a simpler postoperative course compared with traditional anterior lamellar or full-thickness corneal transplantation, while still improving visual quality [98].

Pedrotti et al. evaluated corneal regularization and thickness after meniscus-shaped SLAK in 15 patients with advanced keratoconus. Lenticule diameter and thickness were customized according to each patient’s ectatic area, with a mean lenticule central thickness of 254 ± 114 μm and average gradient of −36 ± 9 μm for each millimetre of diameter toward the periphery. All lenticules were implanted into an intrastromal pocket 100 μm above the endothelium. Then, 3 months postoperatively, an increase in both central corneal thickness and corneal minimum thickness was observed. The anterior Q progressively improved throughout the entire study period, although it did not reach statistical significance. At 12 months postoperatively, significant improvements were observed in the Surface Asymmetry Index (*p* = 0.04), Symmetry Index (*p* = 0.02), and spherical aberration (*p* < 0.001), while changes in coma (*p* = 0.18), HOA (*p* =0.37), and anterior asphericity index (Q) (*p* = 0.31) were not statistically significant. Overall, these findings suggest that customized SLAK’s can improve corneal symmetry and regularity [99]. In a subsequent study, they evaluated corneal biomechanical changes after meniscus-shaped SLAK implantation and reported a significant increase in corneal stiffness represented by higher SP-A1 (*p* < 0.0001), SSI (*p* < 0.0001), and a significant decrease in both 1/R (*p* = 0.01) and DA ratio (*p* < 0.0001). These results are consistent with the expected effects of stromal lenticule addition, which increases geometric stiffness through tissue addition and may further enhance corneal stiffness by inducing a fibrotic response [100].

**Figure 5:**
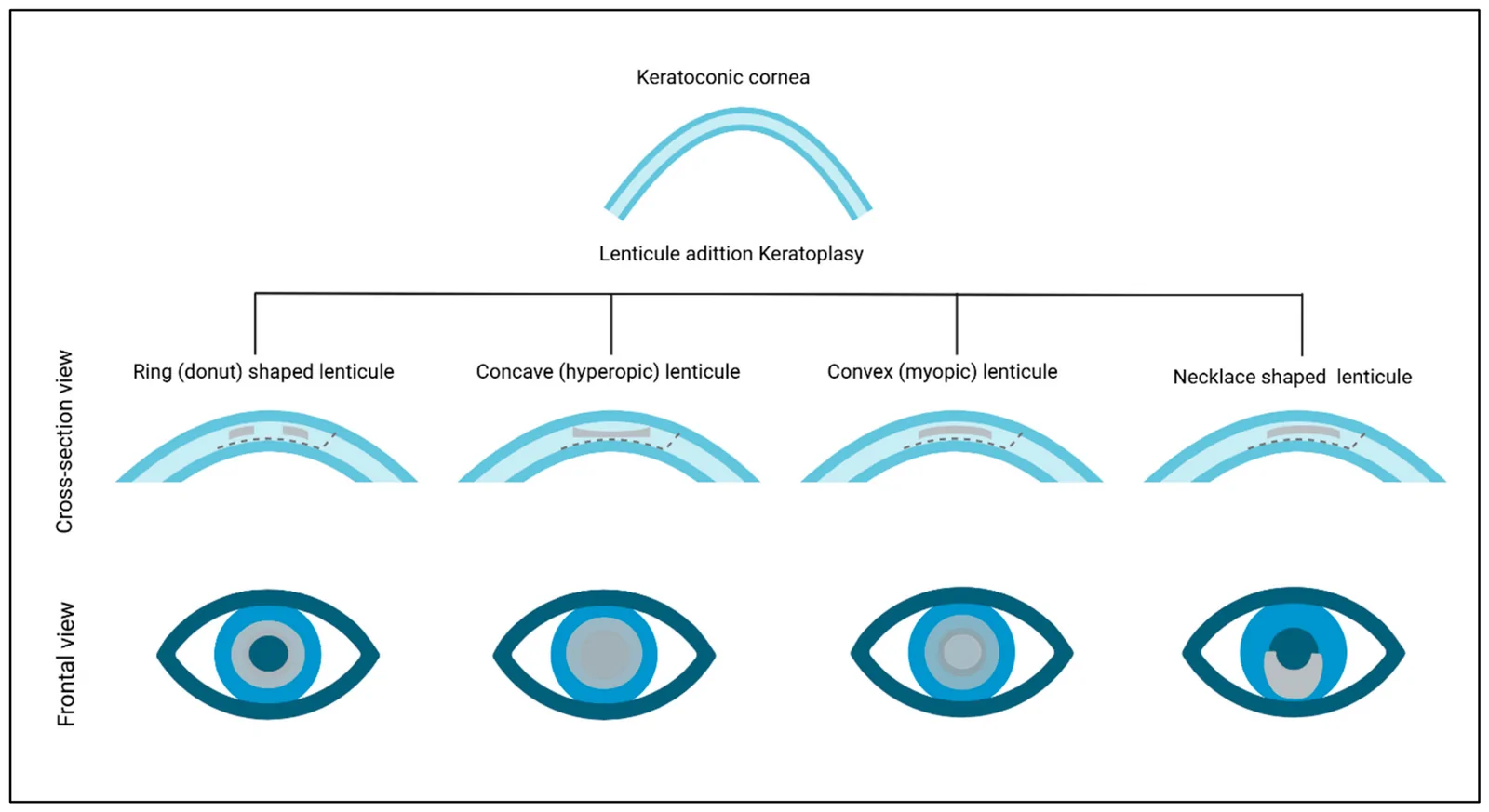
Cross-section (top) and frontal (bottom) view of lenticule implantation with four lenticule shapes: ring (donut) shaped, concave (hyperopic), convex (myopic), and necklace-shaped lenticules. The different configurations aim to achieve different stromal volume augmentation and corneal reshaping customized to the patient’s needs. Figure created in BioRender. Barbaro, V. (2026) https://BioRender.com/a7qop0r. (accessed on 1 September 2026).

More recently, Niazi et al. evaluated the biomechanical effects of customized stromal lenticule implantation in keratoconus. In this prospective study, 22 patients with advanced keratoconus received tailored SMILE-derived lenticules exceeding 100 μm designed to restore corneal thickness and regularity based on individual tomography profiles. Each lenticule was manually customized using 3–5 mm biopsy punches, shaping it into either a “necklace” segment or a 120° ring, depending on the area requiring biomechanical reinforcement (Figure 5). After implantation into a 9.5 mm stromal pocket, significant biomechanical improvements were observed. Mean keratometry decreased from 54.68 ± 2.77 D to 51.95 ± 2.21 D, while SE improved from −13.48 ± 2.86 D to −8.59 ± 2.17 D. Corvis ST showed increased stiffness parameter-A1 from 66.8 ± 13.3 to 100.3 ± 11.4, and central corneal thickness from 301.6 ± 78.1 µm to ~488 µm, along with reductions in integrated radius IR (12.55 ± 2.3 to 10.05 ± 2.09) and highest concavity (HC) deformation amplitude [101]. Overall, these findings provide clinical evidence that KLEx lenticules can be reshaped and tailored to the patient’s needs, thereby improving corneal shape and visual outcomes, as well as reinforcing the biomechanical strength of the ectatic cornea.

**Figure 6:**
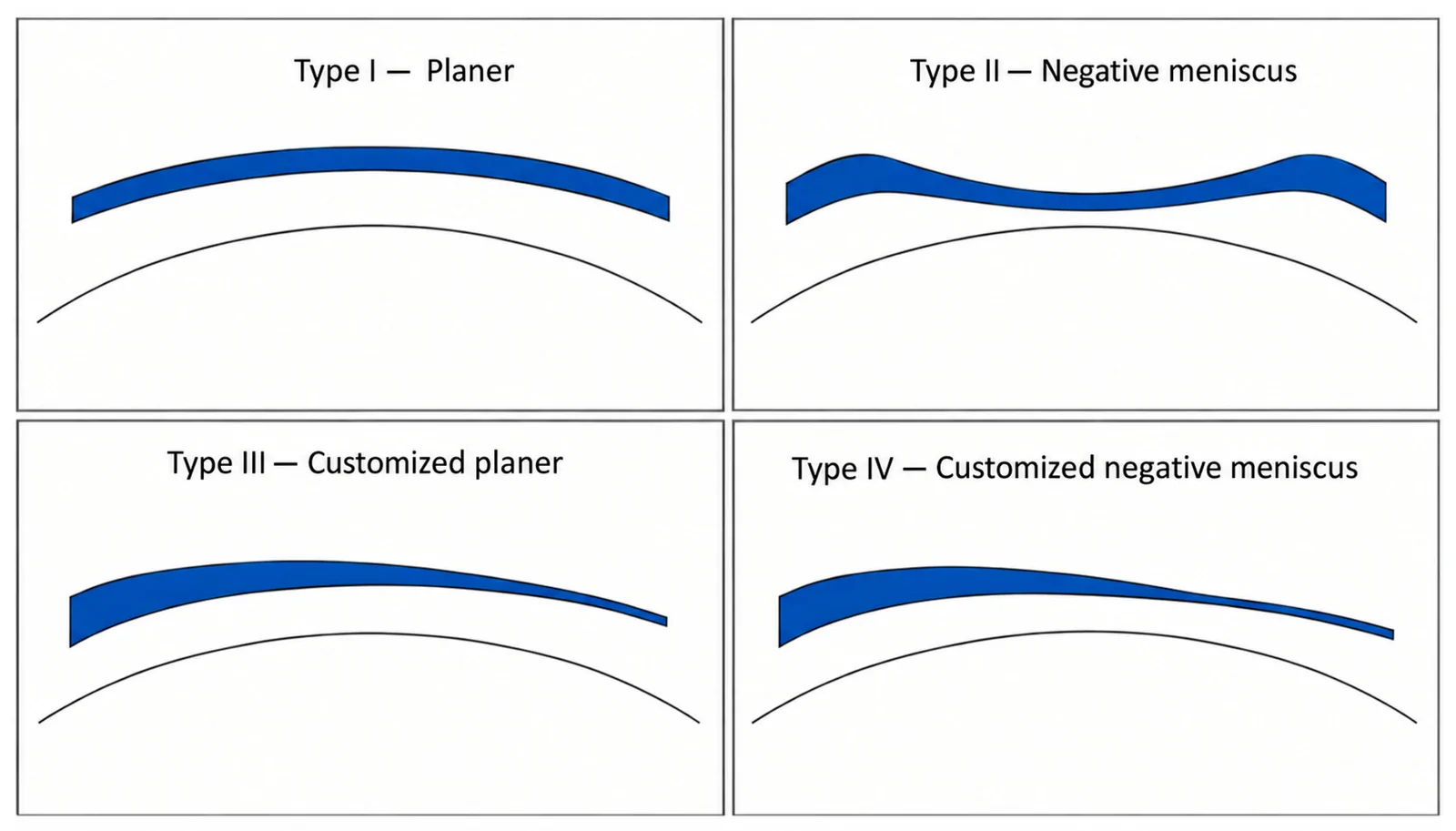
Schematic representation of stromal lenticule profile investigated by Nubile et al. in an ex vivo keratoconus model. Four lenticule profiles were compared: type I—planer (**top right**), type II—negative meniscus (**top left**), and their asymmetrically customized counterparts generated by masked PRK ablation, types III (**bottom right**), and type IV (**bottom left**). Figure Created in BioRender. Barbaro, V. (2026) https://BioRender.com/pckxv8a. (accessed on 1 September 2026).

In a complementary approach, Nubile et al. introduced an ex vivo experimental model of keratoconus to investigate how different excimer laser customized stromal lenticule geometries could optimize SLAK for off-centre ectasia. Twelve donor corneas were reshaped using an excimer laser to reproduce inferiorly decentered steepening and stromal thinning to match real keratoconus topographies. Four lenticule profiles were compared: planar (type I), negative meniscus (type II), and their asymmetrically customized counterparts generated by masked PRK ablation (types III and IV) (Figure 6). Across all implants, cone flattening was consistently observed, with the largest effect achieved by negative and customized negative lenticules. Customized profiles also significantly improved corneal surface regularity and reduced asymmetry, with customized negative lenticules producing the most physiological anterior corneal asphericity. Pachymetric and curvature changes reflected the geometry of each implant, confirming that asymmetric tissue addition, particularly with customized negative lenticules, more effectively redistributed curvature around the cone while limiting undesired peripheral steepening. These results highlight the potential of excimer laser-based lenticule customization to tailor stromal addition to individual cone morphology, broadening SLAK’s applicability beyond centrally located ectasias and paving the way toward personalized corneal reconstruction [102].

In a prospective study, Wei et al. assessed the long-term morphological changes and biomechanical stability of the host cornea in 11 patients with progressive keratoconus over a period of 5 years after small-incision femtosecond laser-assisted lenticule implantation. At 60 months postoperatively, CDVA improved significantly from 1.00 ± 0.19 to 0.48 ± 0.13 logMAR (*p* = 0.026). Corneal densitometry (CD) increased during the first postoperative period, returned to preoperative baseline after 6 months, and remained constant over the following 5 years, indicating recovery of corneal transparency. Biomechanical analysis demonstrated a significant increase in the stiffness parameter at first applanation (SP-A1), from 42.73 ± 8.93 mmHg/mm preoperatively to 80.08 ± 8.94 mmHg/within 1 week postoperatively, with values remaining stable throughout the follow-up period. These findings suggest that this approach provides a steady long-term biomechanical reinforcement while maintaining long-term corneal morphology [103].

Although the preceding studies primarily addressed keratoconus, similar principles have been applied to post-LASIK ectasia. Li et al. evaluated the long-term outcomes of implanting cryopreserved allogeneic lenticules derived from hyperopic SMILE patients to treat post-LASIK ectasia. Six eyes from six patients underwent implantation, which involved lifting the corneal flap, inserting the lenticule, and repositioning the flap, with most cases having prior or simultaneous transepithelial CXL. After at least one year of follow-up, UDVA improved from 1.52 ± 0.40 to 0.74 ± 0.28 logMAR, and CDVA gained one to three lines in all eyes. SE decreased from −14.67 ± 2.36 D to −8.75 ± 4.03 D, accompanied by reductions in anterior keratometry, as five eyes (83.3%) showed a decrease in anterior K values greater than 1.0 D, and none showed an increase greater than 1.0 D [104]. Shortly after, Wong et al. reported the clinical efficacy of SLAK followed by CXL in a case series of five patients with corneal ectasia secondary to FS-LASIK. The primary rationale for adding the stromal lenticule was to increase corneal thickness and allow safe application of CXL. Lenticule refractive power was not deliberately selected, as patients’ refractive error was expected to be corrected with RGP or scleral lens. Postoperatively, corneal thickness range increased from 345 to 404 μm to 413–482 μm, and four out of five patients had improved corneal biomechanical parameters. However, three patients developed irregular astigmatism and transient corneal haze [105].

### 4.2. The Use of KLEx-Derived Lenticules as a Scaffold for Cells

Decellularized corneal scaffolds have gained increasing attention because they provide a more natural environment for the growth and differentiation of cells compared with synthetic alternatives. Specifically, the decellularized stromal lenticule acts as an excellent biological scaffold that can be recellularized with various cell types for therapeutic and experimental purposes. However, the suitability of these scaffolds depends heavily on the decellularization method applied. Different decellularization protocols vary in their ability to balance efficient cell removal and preservation of the native ECM structure of the lenticules [106].

In principle, a decellularized lenticule must meet several criteria: undetectable nuclear material, preserved ECM structure and protein content (including collagen, fibronectin, and glycosaminoglycans (GAG), and good retention of mechanical properties such as elastic modulus and tensile strength [11]. While various decellularization strategies have been reported, including chemical, biological (enzymatic), and physical approaches, they differ in their efficiency in cellular removal and their impact on ECM preservation (Table 3). Alcohol and acids efficiently degrade the cell membrane but risk denaturing ECM components [107,108]. Detergents are effective at disrupting cell membranes but are insufficient for complete removal of cellular material [109,110,111,112]. Enzymes such as phospholipase and trypsin can cause ECM degradation and mechanical weakening [108,113,114,115,116], whereas nucleases (DNase and RNase) are difficult to fully clear and may trigger an immunogenic response upon recellularization [116,117,118]. Physical approaches, such as freeze–thaw cycles [106,118], supercritical carbon dioxide [119,120,121,122], pressurization [123,124], and gamma radiation [125,126,127], require expensive equipment, while ultrasonication and electroporation must be combined with other agents for complete decellularization [128,129] (Table 3). In this context, optimization of decellularization protocols remains a key factor influencing the suitability of KLEx lenticules as scaffolds for corneal tissue engineering.

Alió et al. implanted human stromal sheets (~90 μm thick) recellularized with human adult adipose-derived stem cells (h-ADASCs) into rabbit corneal stroma at 50% depth. After three months, the grafts remained completely transparent with no sign of rejection. Post-mortem analysis demonstrated survival of the transplanted human cells and their differentiation into keratocytes, which was confirmed by human keratocan expression (Table 4) [130]. In a subsequent pilot clinical study, they evaluated decellularized stromal laminae implanted with or without autologous h-ADASC recellularization in patients with advanced keratoconus. While both groups showed improved visual parameters and no adverse effects, recellularization did not provide clear additional benefits. The authors speculated that microstructural disruption during decellularization may have masked potential advantages, leaving the clinical relevance of recellularization uncertain [112].

Aghamollaei et al. investigated the implantation of acellular human SMILE-derived lenticules seeded with Wharton’s jelly mesenchymal stem cells (WJ-MSCs) in a rabbit model. While both acellular and recellularized lenticule implantation showed no signs of rejection, the recellularized lenticules exhibited higher expression of keratocyte markers, specifically CD34, ALDH3A1, lumican, and keratocan, indicating enhanced keratocyte presence and potentially better stromal remodelling and healing [131]. Notably, host cell migration into decellularized lenticules was not observed during the 3-month follow-up, although a small number of keratocytes were detected at the periphery of the grafts. This finding suggests a slow and gradual in vivo recellularization process that may require longer follow-up periods and could be influenced by the absence of chemoattractant signals. In this context, Yam et al. reported the presence of hematoxylin-stained nuclei at the interface between host stroma and implanted lenticules after 5 months, suggesting progressive integration into the host stroma. Re-implantation of SDS-decellularized lenticules in a rabbit model demonstrated good biocompatibility with no signs of necrosis, neovascularization, inflammation, or extrusion, further supporting the suitability of KLEx-derived lenticules as a bioscaffold for stromal tissue engineering. In addition, stromal fibroblasts cultured in vivo on SDS-treated lenticules showed close cellular adherence with negligible cytotoxicity, further indicating their recellularization potential [109].

More recently, Ghiasi et al. advanced this approach by combining decellularized SMILE-derived lenticules with keratocyte-conditioned medium (KCM) to optimize h-ADASC differentiation. This dual strategy significantly increased keratocyte-specific gene and protein expression compared to scaffolds alone. When recellularized lenticules were implanted into rabbit corneas, they remained transparent and well-integrated over 3 months, with no signs of inflammation or neovascularization. Sustained keratocyte-like cell expression was confirmed by PCR and immunohistochemistry. Notably, quantitative assessment of cell survival or density was not performed. Despite this limitation, this study highlights the synergistic effects of extracellular matrix cues and biochemical signalling in stromal regeneration [132].

**Table 3:** Summary of decellularization techniques of KLEx lenticules. Decellularization methods are grouped according to their approach (chemical, biological or physical) and category, and compared according to their mechanism of action, advantages, and limitations.

**Approach**	Category	Agent	Mechanism of Action	Advantages	Limitations	References
Chemical	Alcohols	Ethanol, Acetone	Cell dehydration and lysis through lipid membrane disruption	Simple, rapid, and highly efficient	ECM ultrastructural damage and tissue transparency alteration due to dehydration	Ponce Marquez et al., 2009, Lumpkins et al., 2008 [107,108]
	Ionic detergents	Sodium dodecyl sulfate (SDS)	Solubilization of the cell membrane and denaturation of proteins	Highly effective decellularization	Induced protein denaturation that might affect the ECM organization and protein composition; high cytotoxicity if not fully removed	Yam et al. 2016, Du et al. 2011, Yoeruek et al. 2012, Alio et al. 2018, and Wilson et al. 2016, Marin Tapia et al. 2021 [109,110,111,112,117,133]
	Non-ionic detergents	Triton X-100	Disruption of lipid–lipid and lipid–protein interactions	Minimal disruption of ECM architecture and collagen organization compared to other surfactants	Incomplete removal of cellular and nuclear debris; requires combinations with other agents	Du and Wu, 2011, Wilson et al. 2016, Yam 2016 -decellularization, Marin Tampa et al. 2021 [109,110,117,133]
	Zwitterionic detergents	3-[(3-cholamidopropyl)dimethylamminio]-1 propanesulfonate (CHAPS)	Solubilization of the cell membrane	Better ECM preservation compared to ionic detergents	Poor cellular removal and ECM protein-induced damage; requires combination with other agents	Du et al. 2011, Marin Tapia et al. 2021 [110,133]
	Chelating agents	Ethylene-diamine-tetraacetic acid (EDTA)	Ca^2+^/Mg^2+^ chelating; disrupting cell adhesion and causing cell dissociation	Optical properties retention	Ineffective alone; requires combination protocols	Bayyoud et al., 2012, Oh et al., 2009, Yoeruek et al., 2012, Poornejad et al., 2016, Huh et al., 2018 [111,115,118,134,135]
	Hypotonic and hypertonic solutions	Sodium chloride solution	Disrupt the cell membrane through osmotic shock	Minimal damage to the ECM architecture and proteoglycans (GAG); good optical property retention	Low removal efficacy of cellular and nuclear debris	Gilbert et al., 2006, Yam et al., Wilson et al. 2016, Zang 2015, Gonzales-Andreas et al. 2011 [109,117,136,137,138]
	Acid and basic solutions	Formic acid (HCOOH), Ammonium hydroxide (NH4OH)	Hydrolytic degradation of biomolecules and nucleic acids	Highly effective decellularization	ECM protein damage; altered biomechanics	Choi et al., 2010, Zang 2015, Lin et al. 2019 [137,139,140]
Biological	Esterase	Phospholipase A2	Hydrolysis of ester bonds in phospholipids, contributing to cell membrane degradation	Potentially milder than proteases; supports lipid removal	ECM component disruption	Wu et al., 2009 [113]
	Protease	Trypsin	Proteolytic cleavage of peptide bonds, disrupting cell–matrix adhesions	Efficient cell removal	ECM degradation and weakening if overexposed	He et al. 2016, Huh et al. 2018, Marquez et al., 2009, Procházková, 2024 [108,114,115,116]
	Nucleases	DNase and RNase	Cleavage of phosphodiester bonds in nucleic acids	Minimal damage to the ECM architecture and components	Requires combination with other agents for membrane disruption and access to nucleic acids; difficult to be cleared from the ECM	Wilson et al. 2016, Oh et al., 2009, He et al., 2016 [116,117,118]
Physical	Freeze and thaw		Intracellular ice crystal formation causes mechanical rupture of cell membranes	Simple; no chemical residues; good optical properties compared to chemical detergents	Must be combined with other decellularizing agents to remove DNA remnants from the ECM scaffold	Oh et al., 2009, Crapo et al. 2011 [106,118]
	Supercritical carbon dioxide		Membrane lipid solubilization via supercritical fluid extraction	No toxic residue; simultaneous tissue sterilization; reduced decellularization time	Requires complex ScCO_2_ reactor system	Sawada et al., 2008, Huang et al. 2017, Guler et al. 2017, Liang et al. 2022 [119,120,121,122]
	Pressurization		High hydrostatic pressure (HHP) (>600 MPa) induces irreversible cellular membrane phase transition and protein denaturation.	Efficient decellularization with the maintenance of the collagen fibril matrix	Expensive specialized equipment that generates high HHP (~1GPa) is required	Sasaki et al., 2009, Hashimoto et al. 2010 [123,124]
	Ultrasonication		Acoustic cavitation induces mechanical rupture of the cell membrane	Uniform treatment and short treatment time	May require a combination with detergent (SDS) to complete decellularization	Azhim et al., 2014 [128]
	Gamma radiation		Ionizing radiation induces DNA fragmentation and cellular damage	Simultaneous tissue sterilization; reduced decellularization time	May cause loss of tissue transparency	Henner et al., 1983, Stevenson et al., 2012, Utine et al., 2011 [125,126,127]
	Electroporation		High voltage electrical field induces irreversible nanopore formation and cell lysis	ECM structural preservation	Incomplete removal of cellular and nuclear debris; requires combinations with other agents	Chang et al., 2017 [129]

In addition to stromal application, decellularized KLEx lenticules could also be used as a suitable scaffold for corneal epithelial cells’ survival and proliferation. Hong et al. developed a limbal epithelial stem cell (LESC) carrier by embedding a decellularized KLEx lenticule inside compressed collagen to form a sandwich structure. The biocomposite scaffold demonstrated higher enzymatic stability as well as improved mechanical strength, enabling secure suturing. In a rabbit LSCD model, transplantation of the LESC-seeded biocomposite resulted in stable ocular surface reconstruction with restoration of a stratified corneal epithelium and no evidence of conjunctivalization or neovascularization, supporting potential clinical efficacy [141].

In parallel, Qin et al. seeded induced pluripotent stem cells (iPSCs), differentiated into corneal epithelial-like cells, onto decellularized stromal lenticules. By day 22, immunocytochemical analysis confirmed differentiation by the increased expression of corneal epithelial markers (CK3, CK12, and p63). In addition, the cells exhibited outgrowths and formed a stratified epithelial layer, further supporting the potential of KLEx lenticules as a scaffold for corneal epithelial reconstruction [142].

In a complementary approach, Hazra et al. provided proof-of-concept evidence for using decellularized SMILE lenticules as carriers for human corneal endothelial cells (hCECs). Decellularized SMILE-derived lenticules were incubated in CEC culture medium containing fetal bovine serum (FBS). The scaffold was then constructed by seeding corneal endothelial cells on the lenticules. Immunostaining revealed the formation of a confluent endothelial monolayer expressing ZO-1, Na^+^/K^+^-ATPase, and N-cadherin. When transplanted into oedematous human corneas in an ex vivo model, the construct significantly reduced corneal thickness while maintaining cell viability, demonstrating CEC survival and functionality [143].

Extending beyond the cornea, Gu et al. constructed a retinal pigment epithelium RPE sheet by combining SMILE-derived stromal lenticules with human RPE cells cultured in induced pluripotent stem cell-conditioned medium (iPSC-CM). They reported significant enhancement in RPE cell density, transepithelial electrical resistance (TER), and inhibition of ultraviolet C-irradiated apoptosis when cells were cultured in iPSC-CM. In addition, RPE cells seeded on the lenticule scaffold exhibited morphological and functional features characteristic of native RPE and demonstrated good biocompatibility following subretinal implantation in a rabbit model, highlighting the potential of SMILE-derived lenticules as scaffolds for retinal tissue engineering and future cell-based therapies [144].

Taken together, these studies demonstrate the versatility of KLEx lenticules as scaffolds for a wide range of ocular cell types, supporting their continued development for regenerative ophthalmology and advanced cell-based therapies.

**Table 4:** Summary table of KLEx-derived lenticules as a scaffold for cells. Studies are summarized according to the cell type, experimental model, application, and key findings and reported outcomes.

Study	Cell Type	Experimental Model	Application	Key Finding/Outcomes
Alió et al. (2015) [130]	h-ADASCs	Rabbit	Stromal tissue engineering	Human ADASCs differentiated into keratocytes in vivo; clinical study showed improved vision with no adverse effects, but the recellularization benefit was unclear
Alió et al. (2018) [112]	Autologous h-ADASCs	Clinical		
Aghamollaei et al. (2021) [131]	WJ-MSCs	Rabbit	Stromal tissue engineering	Good graft integration with no signs of rejection; recellularized lenticules showed higher expression of keratocyte markers (lumican, keratocan) compared to acellular control
Yam et al. (2016) [109]	Stromal fibroblast (In vitro)	In vitro + Rabbit (SDS-decellularized lenticule)	Stromal tissue engineering	In vitro: cell adhesion, low cytotoxicity; In vivo: good integration with no signs of necrosis, neovascularization, inflammation or extrusion
Ghiasi et al. (2023) [132]	h-ADASCs + KCM	Rabbit	Stromal tissue engineering	KCM increased keratocyte-specific gene and protein expression; in vivo results showed good lenticule integration, with no signs of inflammation or neovascularization
Hong et al. (2018) [141]	LESCs	Rabbit LSCD model	LSCD (epithelial reconstruction)	The developed collagen-lenticule biocomposite successfully restored stratified epithelium in rabbit LSCD models without neovascularization.
Qin et al. (2019) [142]	iPSCs	In vitro	LSCD (epithelial reconstruction)	Formation of stratified epithelial-like sheets on decellularized lenticules
Hazra et al. (2023) [143]	hCECs	Ex vivo (donor human cornea)	Endothelial Keratoplasty	Proof-of-concept showing a confluent endothelial monolayer; successfully reduced corneal thickness in an ex vivo edema model.
Gu et al. (2019) [144]	RPE cells in iPSC-CM	Rabbit	RPE cell therapy	PSC-conditioned medium enhanced RPE density and barrier function; lenticules supported RPE polarization and maturation; good biocompatibility following subretinal implantation

### 4.3. The Use of KLEx Lenticules as Drug Delivery System

Topical drug administration in the form of eye drops remains the standard and the most common method for the treatment of most ocular diseases [145]. Nevertheless, less than 5% of the applied dose typically remains on the ocular surface long enough to result in a therapeutic effect, primarily due to rapid clearance by blinking and tear turnover [146]. This poor drug retention has driven the development of alternative delivery strategies aimed at extending drug residence time and protecting the active compound from degradation. Among these, drug encapsulation within natural or synthetic micro- and nanoparticles (MPs) has shown promising results in enhancing ocular surface retention and bioavailability [147,148]. More recent innovations include hydrogel- and collagen-based contact lenses, as well as synthetic scaffolds embedded in nanoparticles, which have also demonstrated encouraging results in achieving sustained and localized drug release [149,150,151,152]. Despite these advances, these approaches are not the perfect solution and have their own limitations and side effects, including ocular irritation, blurred vision, hypoxia, and inconsistent retention due to the eye’s intrinsic anatomical and physiological barriers. To address these limitations, the use of the biocompatible and biodegradable human amniotic membrane (hAM) as a drug delivery substrate has recently been proposed [153].

Building on this, recent studies have proposed the use of KLEx lenticules as a natural bio-scaffold suitable for controlled drug delivery. As mentioned earlier, these lenticules exhibit several intrinsic advantages: they are non-immunogenic, mechanically robust, and composed of a well-organized, collagen-rich extracellular matrix (ECM) obtained from young, healthy donors. Notably, the ECM acts as a reservoir for growth factors (GFs), as proteoglycans within the matrix contain GF-binding domains. Additionally, structural proteins such as collagen and fibronectin include integrin-binding motifs (e.g., the RGD sequence, Arg-Gly-Asp) which interact with the cell surface integrin receptor. Together, these molecular features allow ECM-based scaffolds to coordinate integrin and GF receptor signalling, thereby enhancing and prolonging cellular responses [22,154,155,156].

**Figure 7:**
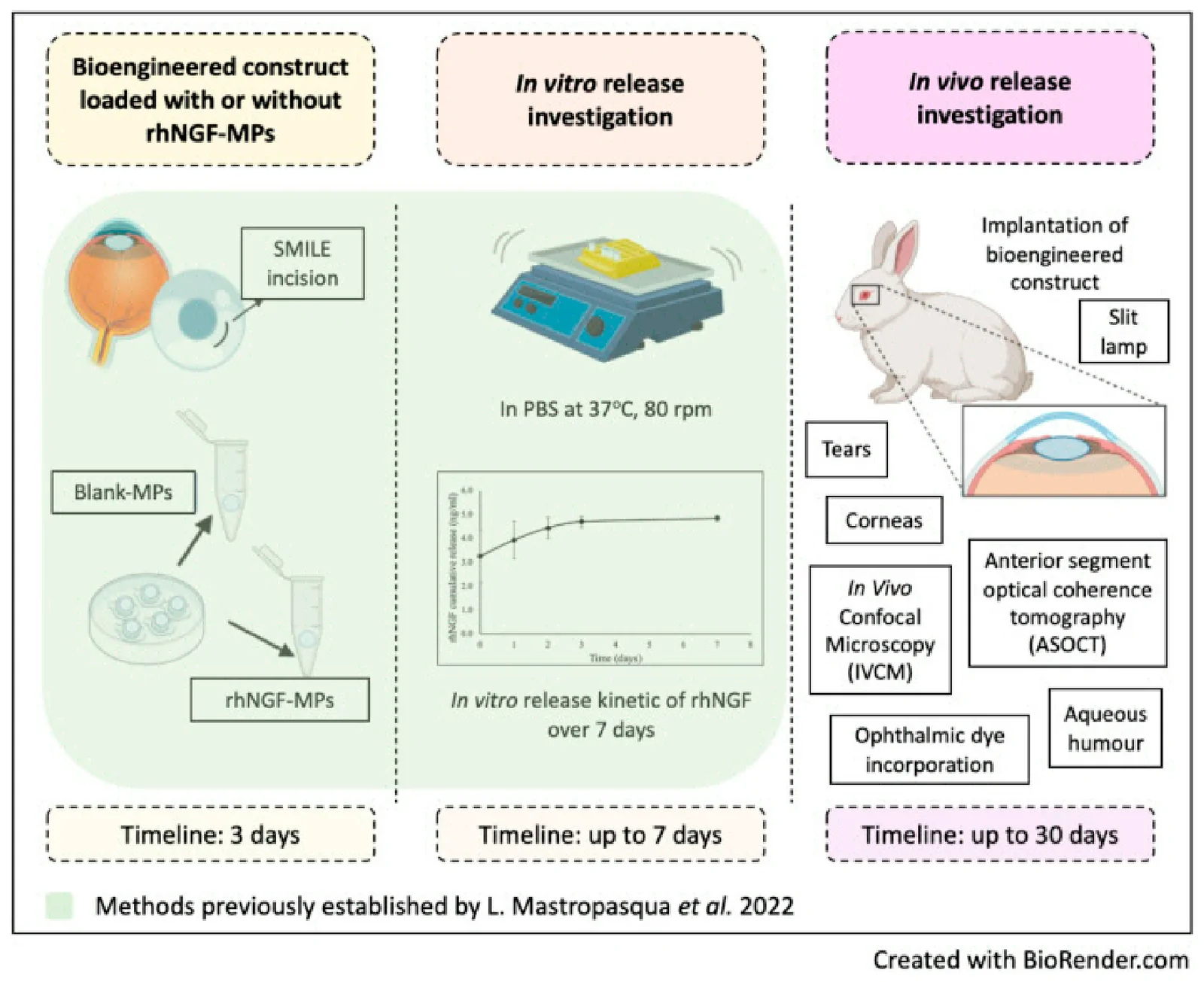
Schematic diagram outlining the methods implemented in the study of Mastropasqua et al. [157] and Lin et al. [158]. Reprinted from Lin et al. (2025) [158] with permission.

In this context, Mastropasqua et al. investigated the use of SMILE-derived stromal lenticule as an ocular drug delivery system for Nerve Growth Factor (NGF). Decellularized lenticules were incubated with polylactic-co-glycolic acid (PLGA) microparticles (MPs) loaded with recombinant human NGF (rhNGF-MPs), followed by kinetic release analysis. The authors demonstrated that PLGA-MPs were effectively incorporated both on the lenticule surface and within the stromal matrix. In vitro experiments showed a rapid initial burst within the first 24 h, followed by sustained rhNGF release for up to one month, with preserved biological activity [157]. It is important to note that while the lenticule was safe to use in its native form, decellularization further enhanced biocompatibility, reduced immunogenicity, and created stromal micropores that facilitated deeper MP incorporation. Recently, the same group expanded this framework and investigated the translational potential of rhNGF-MPs sustained release through in vitro and in vivo evaluation. In accordance with the previous findings, an initial rapid in vitro release of rhNGF was detected. In vivo results demonstrated that rhNGF-MP-loaded lenticules were biocompatible, with no signs of corneal inflammation, edema, or infiltration at the implantation site throughout the entire follow-up period. Furthermore, the in vivo release profile indicated a rapid release in the first two days, consistent with the ex vivo finding, followed by a sustained release for up to 1 month. The authors explained that the discrepancy observed in the release profile between the two studies could be attributed to differences in lenticule source (donor variability) and rhNGF MP batch properties. In addition, in vivo findings exhibited a significantly higher NGF level in the corneal tear of the rhNGF-MP group compared with the negative control. Notably, a significant increase in corneal nerve fibre density was observed at week 3 in the rhNGF-MP group compared with the Blank-MP group, supporting their potential role in nerve regeneration capacity (Figure 7) [158].

Pelusi et al. investigated the use of a bioengineered lenticule scaffold for the co-delivery of cellular and protein-based therapeutics. They evaluated the effects of human amniotic fluid stem cells (hAFSCs) and recombinant human NGF (rhNGF), delivered by bioengineered lenticules, on a high-glucose-induced ex vivo model of diabetic retinopathy using porcine neuroretinal explants. Their main finding was that lenticules bioengineered with both hAFSCs and rhNGF-loaded microparticles produced a synergistic effect, significantly reducing the expression of inflammatory, oxidative, apoptotic, and angiogenic markers while simultaneously increasing key retinal markers [27]. This proof-of-concept study successfully demonstrates the potential of the lenticule as a multifaceted delivery system for complex therapies. The above-mentioned findings are particularly encouraging in light of the current limitations associated with NGF delivery. Topical cenergermin (Oxervate^®^) requires administration six times per day for 8 weeks, which may negatively affect the patient’s adherence. In addition, the drug represents a logistical challenge, as it must be transported and stored in a frozen state, further complicating its clinical use. Contrary to the decellularization-induced pore formation described previously, EDC/NHS crosslinking created a more compact lenticule, potentially modifying its drug loading and release mechanism. Indeed, the crosslinked lenticules demonstrated a sustained release of the antibiotic levofloxacin over 21 days in vitro. The extended release translated to potent and prolonged antibacterial activity against *Staphylococcus aureus*, with the optimally crosslinked group (0.05 mmol EDC/mg) exhibiting the highest drug release and largest zone of inhibition [159]. This study emphasized the versatility of the lenticule scaffold, demonstrating that its properties can be tuned via different bioengineering techniques, either by creating pores for microparticle incorporation or by crosslinking to enhance intrinsic drug loading for tailored long-term ocular drug delivery applications.

Rao et al. evaluated the sustained-release capacity and antibacterial activity of decellularized and crosslinked SMILE-derived lenticule, loaded with levofloxacin. Crosslinking was performed using different concentrations of Carbodiimide (EDC) or N-hydroxysuccinimide (NHS), in order to identify the optimal crosslinking approach. In contrast with previously described decellularization-induced pore formation, the rationale behind lenticule crosslinking was to reduce the gaps between the collagen fibres and create a more compact scaffold in order to achieve sustained and prolonged drug retention and release. Results demonstrated sustained in vitro levofloxacin release over 21 days. Among the tested groups, 0.05 mmol EDC/mg lenticule (E/L05) exhibited the highest drug release and the largest antibacterial zone of inhibition against *Staphylococcus aureus*. The highest released drug concentration was 233.02 ± 47.52 µg/mL, exceeding the MIC while remaining below toxicity levels. Notably, excessive crosslinking likely produced an overly compact collagen network, limiting drug uptake and consequently reducing antibacterial efficacy. Overall, this study highlights the versatility of lenticule scaffolds, demonstrating possible tuning of drug-releasing properties through different bioengineering techniques [159].

Recently, the same group assessed the drug release capability of crosslinked and decellularized SMILE-derived lenticules loaded with silver nanoparticles (AgNPs), a potent broad-spectrum antimicrobial. Their results showed that AgNP-loaded lenticules maintained high optical transparency (>90% transmittance at 555 nm) as well as improved structural stability. In addition, released AgNP concentrations exceeded the minimum inhibitory concentration for *Staphylococcus aureus* and *Pseudomonas aeruginosa* [160]. Unlike Mastropasqua’s NGF study, AgNP release did not follow a prolonged release profile, but a rapid burst with complete release occurring within the first hour. Despite this fast release, this work highlights the potential of bioengineered lenticules not only for delivering biological factors but also as antimicrobial patches in cases of infectious keratitis where immediate management is critical [160].

### 4.4. The Use of KLEx Lenticule for Tectonic Keratoplasty

#### 4.4.1. Biological Patches for Corneal Perforation and Macular Hole

Corneal perforation is a severe outcome of various infectious and non-infectious disorders, including bacterial, fungal, and herpes keratitis, trauma, and immune disorders. It requires urgent intervention in order to preserve the integrity of the eye globe, control inflammation or infection, and prevent further complications. Management depends largely on the size and location of the perforation as well as the underlying etiology. Treatment ranges from temporary measures, including adhesive gluing, conjunctival flaps, scleral lamellar graft, and amniotic membrane transplantation, to more prominent interventions, such as corneal transplantation [161]. However, conventional temporary approaches often prove inadequate for perforations larger than 2.0 mm or those associated with significant stromal tissue loss [162,163]. While keratoplasty remains the gold standard for definitive repair, its application is limited due to the worldwide shortage of donor tissue [20]. This is particularly critical in developing countries, where many patients are unable to access donor corneas. Moreover, corneal grafting requires suturing, which can cause various complications, including postoperative astigmatism, epithelial ingrowth, graft rejection, and even graft failure, in addition to prolonging the surgical time and patient discomfort. Collectively, these limitations highlight the need for alternative, readily available, and effective temporary solutions for the emergency management of corneal perforation. In this context, increasing evidence supports the use of KLEx lenticules as biological patches. In addition to providing immediate mechanical support and corneal sealing, these lenticules act as a biocompatible stromal scaffold, potentially promoting wound healing and host cell repopulation, therefore representing a promising option for temporary closure and structural reinforcement of corneal perforations [164,165].

The first *clinical* use of SMILE-derived lenticules to treat perforation was reported by Wu et al., who described the use of SMILE-derived lenticules in a case series of six patients with corneal perforation. In this study, two oversized lenticules were sutured over the perforation site. All eyes were successfully sealed, and three patients (50%) demonstrated postoperative improvement in BCVA. Partial stromal integration with complete re-epithelialization was observed within 3–4 weeks, and no recurrence of infection or perforation occurred during the 12-month follow-up (Table 5). Notably, partial dissolution of the lenticule edges was observed approximately two weeks postoperatively. The authors speculated that this could be due to proteolytic degradation mediated by inflammatory matrix metalloproteinases (MMPs), bacterial proteases, or lysosomal enzymes released during apoptosis. Moreover, the thinness of the lenticule edge (minimum ~15 µm) likely made it more susceptible to such enzymatic breakdown [166]. In contrast, El Aziz et al. later demonstrated that comparable outcomes could be achieved using a simplified construct consisting of a single-layer stromal lenticule covered by a single-layer amniotic membrane. In their series of seven patients, complete anatomical closure was obtained in all cases, with postoperative BCVA improvement in 42.9% of eyes and no recurrence or reperforation reported during follow-up [29].

Jiang et al. extended these observations with a larger case series involving 20 patients (22 eyes) with corneal ulcer or perforation. Trimmed SMILE-derived lenticules were sutured to the host cornea, without the use of an overlying amniotic membrane, resulting in restoration of globe integrity in all cases and a significant improvement in mean BCVA from 0.17 ± 0.20 to 0.27 ± 0.25. No immune rejection or recurrent perforation was observed. Nevertheless, in three cases, repeated surgery was required at 3 months due to residual corneal thickness < 250 μm [167].

While suturing proved effective, subsequent studies explored alternative fixation methods to simplify the procedure and reduce suture-related complications. Bhandari et al. were the first to report a sutureless technique, using human fibrin glue to secure SMILE-derived lenticules in seven eyes with corneal microperforations or complex corneal tears. Lenticules were customized to the defect geometry, and all grafts remained well apposed and clear throughout follow-up. Visual improvement was observed from 15 days onward postoperatively in the majority of cases, demonstrating the feasibility of adhesive-based fixation [168].

For severe macroperforations requiring additional structural support, Jacob et al. described a hybrid technique combining initial suturing of the perforation edges with placement of a SMILE-derived lenticule secured using fibrin glue. This technique successfully resolved a double anterior chamber and resulted in stable visual recovery, with graft clarity maintained at 18 months, highlighting the versatility of lenticules as reinforcing biological patches [169].

Non-infectious perforations, such as those associated with trauma or Mooren ulcer, generally cause minimal inflammatory response with limited tissue infiltration. In these cases, the use of the KLEx lenticules patch may therefore be particularly advantageous. Yang et al. reported a clinical series comprising 17 eyes with various corneal perforation sizes ranging from 1 to 2 mm, with different etiologies, including bacterial, fungal, and herpes infection, corneal dermoid tumour, Mooren ulcer, and trauma. SMILE-derived lenticules were trimmed and adjusted to the size and shape of the perforation and implanted directly on the affected area, followed by suturing. The gap between the implanted lenticule and the recipient stroma was filled with an additional small lenticule in order to reduce postoperative astigmatism and improve uncorrected visual acuity. During the 6-month follow-up, no adverse events were detected, and complete corneal re-epithelialization was achieved within 2 weeks postoperatively. In addition, significant improvements in CDVA were observed in 8 of 17 eyes. However, nine eyes did not show any significant change in visual acuity [170].

Extending earlier multilayer patching concepts, Kotb and Elsayed et al. evaluated a stacked double-lenticule configuration in eyes with impending corneal perforation. In their series of 20 eyes, two stromal lenticules (approximately 65 µm each) obtained from different donors were stacked to create a thicker tectonic graft (mean thickness ~109 µm) and sutured over the area of thinning. This approach restored globe integrity in 19 patients (95%), prevented progression to full-thickness perforation in 16 (80%), and 15 patients (75%) exhibited a statistically significant improvement in visual acuity. No immune rejection occurred in any patient. However, two cases showed persistent hypotony and were managed by amniotic membrane graft augmentation, and one case exhibited continuous leak with progressive infection and ended with penetrating keratoplasty [171].

In a comparative retrospective study of 40 eyes with medium-sized perforations, Tawfeek et al. reported that both SMILE-derived lenticules and amniotic membrane grafts augmented with platelet-rich plasma achieved complete closure. Notably, healing was significantly faster in the lenticule group, highlighting the potential advantage of SMILE-derived grafts in promoting rapid corneal repair [172].

More recently, Klimsova et al. reported the use of SMILE-derived lenticules in 12 complex eyes, including cases with prior failed amniotic membrane transplantation for the treatment of corneal ulcers. In three cases, lenticules were secured with interrupted sutures, whereas in the remaining eyes, the sutureless approach was adopted using an intrastromal pocket. In all but one case, a supplementary amniotic membrane was sutured over the lenticule. Successful sealing and re-epithelialization were achieved in seven eyes (58%), with no signs of infection or immune rejection observed during follow-up. However, three eyes required subsequent PK, and in two eyes, a scleral patch surgery was performed. Notably, the use of cryopreserved lenticules in this study highlights their potential in overcoming tissue availability and logistical constraints [173].

This progression toward more refined surgical strategies was further advanced by the “sandwich” approach described by Chen et al., in which a trephined, marked lenticule (~6.0 mm) was inserted into a mid-stromal pocket and secured with two sutures in nine eyes. Anatomical sealing and anterior chamber restoration occurred immediately in all cases. During follow-up, no epithelial ingrowth, infection, or rejection was observed, and none of the patients required keratoplasty; OCT confirmed stable graft integration (mean thickness 122 ± 21 μm) and clear interfaces. At 6 months, mean BCVA improved significantly from 0.18 ± 0.12 to 0.52 ± 0.31 logMAR (*p* < 0.001), along with reductions in refractive error and keratometry [174].

Recently, Mergen et al. reported a retrospective study involving 16 eyes from 16 patients who underwent SMILE-derived lenticule patch graft implantation for the treatment of corneal perforation ranging from 0.5 to 4 mm in size. Underlying aetiologies included neurotrophic keratitis, penetrating injuries, and corneal melting due to inflammatory or rheumatological disorders. At 1 year postoperatively, no evidence of graft rejection or failure was observed, and corneal integrity was achieved in all cases. Mean logMAR BCVA improved from 1.9 ± 0.8 preoperatively to 1.2 ± 0.7 postoperatively. Additionally, amniotic membrane transplantation was performed in 3 cases, while PK was required in 4 cases in order to improve BCVA. Notably, all the patients who underwent PKP had full-thickness defects, suggesting that this approach might be less favourable in larger and deeper corneal perforations [175].

Expanding beyond the cornea, recent studies investigated the use of KLEx lenticule for macular hole repair. Initially, pars plana vitrectomy combined with internal limiting membrane (ILM) peeling and silicone oil tamponade represented the standard treatment for macular hole retinal detachment, particularly in high myopic eyes [176]. However, due to surgical complexity and challenges related to retinal floatation and the thin, fragile nature of the ILM, various surgical techniques were developed, including lens capsular flap transplantation [177], amniotic membrane transplantation [178], and autologous neurosensory retinal transplantation [179]. These grafts act as a scaffold that promotes cell proliferation and facilitates retinal layer regeneration. Although these approaches yielded satisfactory anatomical outcomes, they carry the risk of potential complications such as retinal surface irregularities, parafoveal atrophy, choroidal neovascularization, and retinal detachment [180,181,182]. In addition, autologous neurosensory retinal transplantation is a relatively complex procedure and possibly redundant, given that there are simpler surgical alternatives with comparable outcomes. In light of these limitations, corneal stromal lenticule transplantation could offer an alternative, simpler surgical approach for macular hole repair, as they are readily available, easier to handle intraoperatively, and less technically demanding. Recently, Ding et al. reported successful sealing of macular holes in three eyes treated with SMILE lenticule transplantation. Moreover, improved visual acuity was achieved, and no adverse events were detected during follow-up [183].

Shortly after, Zhang et al. reported the use of stromal lenticules combined with C3F8 gas or silicone oil (SO) tamponade for the treatment of recurring macular holes and macular hole retinal detachments in 15 patients who had previously undergone vitrectomy and ILM peeling. At 6 months postoperatively, macular hole closure and anatomical retinal reattachment were achieved in all cases. In addition, logMAR BCVA significantly improved from 1.95 ± 0.27 preoperatively to 1.33 ± 0.21 postoperatively (*p* < 0.05), and no displacement of the stromal lenticule was detected. Furthermore, optical coherence tomography angiography (OCTA) revealed a gradual decrease in the foveal avascular zone (FAZ) and a parallel gradual increase in superficial vascular density (SVD), indicating improved microvascular macular blood flow. Despite the high success rate in achieving anatomical closure and improved visual impairment, future studies with longer follow-up periods are necessary to investigate the long-term remodelling and absorption pattern of stromal lenticule beyond 6 months [184].

#### 4.4.2. KLEx-Derived Lenticules for Pterygium and Dermoid Treatment

Pterygium is a chronic inflammatory lesion that develops on the bulbar conjunctiva, typically in the nasal canthal region of the palpebral fissure [185]. Although several surgical techniques are available for its excision, recurring pterygium remains challenging to manage, as repeated re-excision surgeries may induce corneal thinning and scarring, which may eventually lead to perforation and compromised visual outcomes [186,187]. In this context, tectonic keratoplasty using KLEx lenticules could offer a promising therapeutic alternative to overcome these challenges by providing structural reinforcement to areas of stromal thinning.

**Figure 8:**
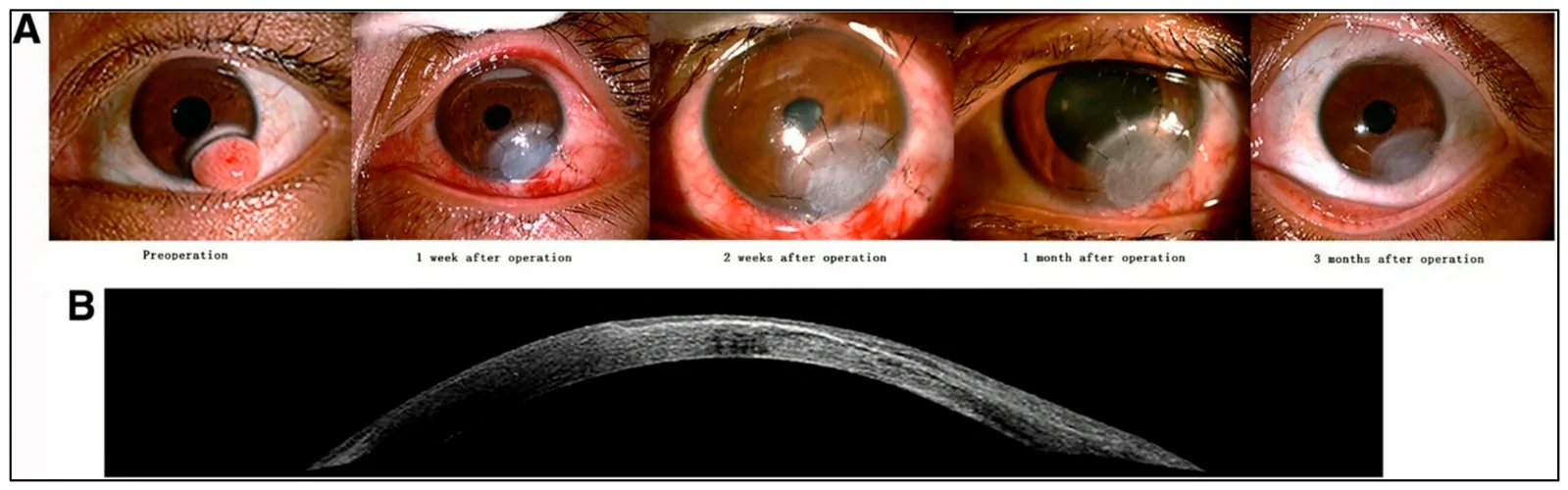
Clinical outcomes of lenticule implantation for limbal dermoid reconstruction. (**A**) Preoperative and postoperative external eye image of a patient with limbal dermoid. (**B**) Anterior segment OCT image showing the donor lenticule and recipient bed and the donor lenticule with overlying epithelium. Reprinted from Wan et al. 2020 [191].

Pant et al. reported a successful use of a SMILE-derived lenticule combined with a single layer of amniotic membrane in a patient with recurrent pterygium complicated by thin cornea, who had previously undergone three bare sclera pterygium excisions. Complete epithelialization was achieved 1 week postoperatively, and the graft remained intact with no signs of rejection throughout the 8-month follow-up period [188]. More recently, Mutlu et al. demonstrated the efficacy of this approach in patients with primary pterygium. No rejection, complications, or recurrence in any of the cases were reported during the 6-month follow-up period, further indicating that Tectonic keratoplasty using stromal lenticule is an effective, safe, and cost-effective method for the management of corneal thinning in pterygium [189]. Similarly, KLEx-derived lenticules have been used for the management of corneal dermoid. Studies demonstrated fast healing and re-epithelialization, tectonic integrity preservation, and improved visual acuity following dermoid excision and lamellar keratoplasty using stromal lenticule (Figure 8) [190,191,192,193].

#### 4.4.3. The Use of KLEx Lenticules as a Patch Graft in Glaucoma Drainage Implant Surgery

Glaucoma drainage device surgery (GDDS) has become a common procedure in the management of glaucoma [194]. However, conjunctival erosion and tube exposure remain a major complication, accounting for approximately 5–10% of the cases [195,196]. Different materials have been used to cover the drainage tube, including sclera, pericardium, dura mater, amniotic membrane, and femoral fascia [197,198,199]. Encouraging results have been reported with the use of donor stromal tissue for the management of tube exposure. Additionally, its dense collagen structure reduces the risk of tissue melting [200,201,202]. Consequently, several studies evaluated the use of KLEx lenticles for the management and repair of drainage tubes [203,204,205,206]. Notably, in a comparative study between allogeneic scleral and stromal patch grafts for the prevention of tube exposure, no statistical difference in recurrence or clinical efficacy was found. However, KLEx lenticules offer a cosmetic advantage due to their thin and transparent nature, especially when the surgical site is at the inferior conjunctiva [204]. Furthermore, a double-layer approach with KLEx lenticules could be advantageous as it can achieve adequate coverage and reduce the risk of exposure and graft rejection [206]. Of note, crosslinking of the graft prior to implantation could also offer an advantage, as it would enhance stiffness and thereby increase resistance to erosion [205].

## 5. Conclusions and Future Outlook

KLEx lenticules represent a valuable source of transparent, biocompatible stromal tissue that can be repurposed and applied for a wide range of refractive and therapeutic applications. Their growing clinical relevance reflects the broader shift in regenerative ophthalmology towards reversible additive corneal surgery rather than tissue removal, which represents a more mechanically challenging approach. Despite the promising results, clinical use of KLEx lenticules remains limited, and further refinement of surgical planning and standardized nomograms is required in order to improve accuracy and reduce variability. Moreover, further studies are needed to attest to the efficacy and long-term stability of this approach.

With continued progress in laser reshaping and tissue bio-engineering techniques, lenticules could potentially be precisely modified and reshaped according to specific clinical needs [207]. Customization may be further enhanced using AI-tomography-guided tools, enabling patient-specific lenticule shapes tailored to individual patients’ needs. From a practical perspective, the widespread application of KLEx lenticules heavily depends on the establishment of a standardized KLEx lenticule banking system. Despite the promising results and the growing popularity, there is still no standardized banking system to facilitate distribution and global access. This is particularly relevant in regions with limited access to donor corneas. Therefore, future progress will likely depend on a multidisciplinary collaboration between refractive surgery centres and eye banks, while carefully applying regulatory requirements and quality assurance measures. Overall, this review highlights the versatile applications of KLEx lenticules and emphasizes the role of eye banks in facilitating their broader clinical adaptation, reducing dependence on donor corneas, and expanding access to corneal regenerative therapies.

## Figures and Tables

**Table 5:** Summary of clinical outcomes of KLEx lenticules as biological patches for corneal perforation. Studies are summarized according to number of eyes treated, surgical technique and clinical outcomes. ^°^ Case report; # case series; ^Δ^ clinical study; * retrospective study.

Study	Number of Eyes Treated	Surgical Technique	Clinical Outcome
^Δ^ Wu et al. (2015) [166]	6	2 oversized lenticules sutured over the perforation. An extra AM patch was applied in 5 patients.	100% sealing; 3 patients (50%) had improved postoperative BSCVA; re-epithelialization in 3–4 weeks; no infection or relapse.
^Δ^ Elaziz et al. (2017) [29]	7	1 oversized lenticule sutured over the perforation with an overlying layer of AM.	100% sealing; 3 patients (42.9%) had improved postoperative BSCVA; no infection or reperforation.
^#^ Jiang et al. (2016) [167]	22	Trimmed lenticule sutured over the perforation.	100% globe integrity; mean BCVA improved from 0.17 ± 0.20 to 0.27 ± 0.25; no immune rejection or perforation; 3 cases required repeated surgery due to residual corneal thickness < 250 μm.
^#^ Bhandari et al. (2016) [168]	7	Customized lenticule patch secured with fibrin glue (sutureless technique).	100% patch graft acceptance with re-epithelialized surface; significant improvement in CDVA in 5 eyes; no incidence of graft displacement, aqueous leakage, additional stromal lysis, immune rejection, or neovascularization.
^°^ Jacob et al. (2019) [169]	1 (case report)	Hybrid technique: lenticule sutured with fibrin glue-assisted closure.	Successful sealing, resolution of double anterior chamber, and vision recovery; stable outcome with clear graft at 18 months.
^#^ Yang et al. (2020) [170]	17	Customized lenticule patch sutured over the perforation, followed by an additional lenticule filling the gap between the graft and the recipient bed.	No adverse events were detected, and complete corneal reepithelialization was achieved within 2 weeks postoperatively. Significant improvements in CDVA were observed in 8 of 17 eyes.
^Δ^ Kotb and Elsayed et al. (2022) [171]	20	Double-stacked lenticular suture.	95% globe integrity; improvement in visual acuity in 75%; 2 cases required AM graft augmentation; 1 case required subsequent PK.
* Tawfeek et al. (2023) [172]	40	Lenticule vs. AM with platelet-rich plasma (comparative study).	100% closure in both groups; significantly faster healing in the lenticule group.
* Klimesova et al. (2024) [173]	12	Cryopreserved lenticule implantation: sutured fixation (n = 3) or sutureless intrastromal pocket (n = 9). An overlay amniotic membrane (sutured) was applied in 11 cases.	58% achieved sealing with re-epithelialization with no infection or rejection; 3 eyes underwent subsequent PK, and 2 eyes required scleral patch surgery.
^Δ^ Chen et al. (2024) [174] NCT06233409	9	Intrastromal lenticule insertion (“sandwich” technique).	100% sealing and anterior chamber restoration occurred immediately; BCVA improved from 0.18 ± 0.12 to 0.52 ± 0.31; no epithelial ingrowth, infection, or rejection.
^Δ^ Mergen et al. (2025) [175] NCT06233409	16	Lenticule patch graft with sutures	No evidence of rejection, graft rejection, or failure was observed in any patient; Amniotic membrane transplantation was performed in 3 patients, and PK in 4 patients.

## Data Availability

The data supporting the findings of this study are available from the corresponding authors upon reasonable request.
